# SCARECROW is deployed in distinct contexts during rice and maize leaf development

**DOI:** 10.1242/dev.200410

**Published:** 2022-03-30

**Authors:** Thomas E. Hughes, Jane A. Langdale

**Affiliations:** Department of Plant Sciences, University of Oxford, South Parks Road, Oxford, OX1 3RB, UK

**Keywords:** SCARECROW, Rice, Maize, Stomata, Patterning

## Abstract

The flexible deployment of developmental regulators is an increasingly appreciated aspect of plant development and evolution. The GRAS transcription factor SCARECROW (SCR) regulates the development of the endodermis in *Arabidopsis* and maize roots, but during leaf development it regulates the development of distinct cell types; bundle-sheath in *Arabidopsis* and mesophyll in maize. In rice, SCR is implicated in stomatal patterning, but it is unknown whether this function is additional to a role in inner leaf patterning. Here, we demonstrate that two duplicated SCR genes function redundantly in rice. Contrary to previous reports, we show that these genes are necessary for stomatal development, with stomata virtually absent from leaves that are initiated after germination of mutants. The stomatal regulator OsMUTE is downregulated in *Osscr1;Osscr2* mutants, indicating that OsSCR acts early in stomatal development. Notably, *Osscr1;Osscr2* mutants do not exhibit the inner leaf patterning perturbations seen in *Zmscr1;Zmscr1h* mutants, and *Zmscr1;Zmscr1h* mutants do not exhibit major perturbations in stomatal patterning. Taken together, these results indicate that SCR was deployed in different developmental contexts after the divergence of rice and maize around 50 million years ago.

## Abstract (precis)

**Summary:** The transcription factor SCARECROW patterns stomata in rice leaves, whereas in maize it predominantly patterns the inner leaf.

## INTRODUCTION

The coordination of cell patterning is fundamental to multicellularity. In plants, leaf function is underpinned by the correct spatial specification of an array of specialised cell types. The inner leaf consists of photosynthetic mesophyll cells and a network of vasculature with associated bundle-sheath cells, whereas the leaf surfaces comprise epidermal pavement cells, stomatal pores that regulate gas exchange across the leaf surface and hair cells. In grasses, parallel veins within the leaf are flanked by files of stomata in the epidermis (Stebbins and Shah, 1960), requiring coordinated development between the inner and outer leaf layers (McKown and Bergmann, 2020).

The GRAS transcription factor SCARECROW (SCR) is one of the best understood plant developmental regulators, having first been identified through its role in regulating cell-type patterning in roots (Laurenzio et al., 1996). In this context, *SCR* is expressed in the initial cells (Laurenzio et al., 1996) that divide asymmetrically to form endodermal and cortical cell-layers around the stele (Dolan et al., 1993). In the absence of SCR, this asymmetric cell division does not occur, resulting in a mutant cell-layer with features of both the endodermis and cortex (Laurenzio et al., 1996). A similar phenotype is seen in maize, where *Zmscr* mutants fail to properly specify the endodermis (Hughes et al., 2019). Furthermore, both *Arabidopsis* and maize *scr* mutants exhibit a perturbed growth phenotype (Dhondt et al., 2010; Hughes et al., 2019; Hughes and Langdale, 2020). In contrast to these conserved functions, SCR has divergent functions in leaves: patterning bundle-sheath cells around veins in *Arabidopsis* (Cui et al., 2014; Wysocka-Diller et al., 2000) but regulating mesophyll cell specification and division in maize (Hughes et al., 2019).

**Fig. 1.**
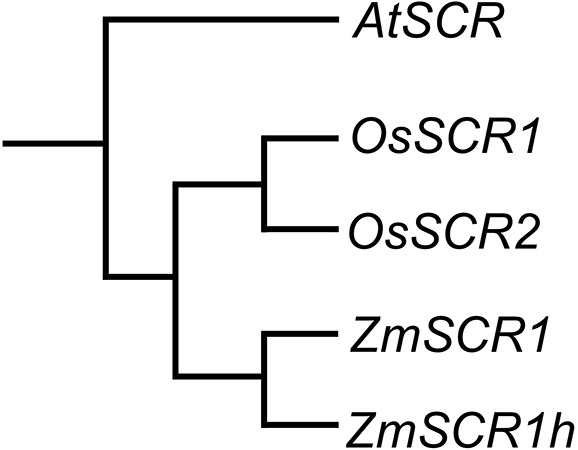
**Cartoon phylogeny showing the relationships between *Arabidopsis* (*At*), rice (*Os*) and maize (*Zm*) SCR genes.** Phylogeny taken from Hughes et al. (2019).

Asymmetric cell divisions are a common feature of many plant developmental pathways, including stomatal patterning. In eudicots, a meristemoid mother cell divides asymmetrically to give rise to a meristemoid, which can then be specified as a guard mother cell (GMC). In monocots, where stomata develop in rows flanking parallel veins, once the stomatal cell file is established, cells divide asymmetrically to form a larger interstomatal sister cell and a GMC (Stebbins and Shah, 1960; McKown and Bergmann, 2020; Nunes et al., 2020). In both cases, once established, the GMC differentiates and divides symmetrically into the stomatal guard cell pair (Conklin et al., 2019; McKown and Bergmann, 2020; Nunes et al., 2020; Guo et al., 2021). Consistent with its role in asymmetric cell divisions in root development, it has been suggested that SCR may be required for stomatal patterning in rice (Kamiya et al., 2003; Wu et al., 2019). Unlike in maize, where *ZmSCR* transcripts accumulate primarily in the inner leaf during development (Hughes et al., 2019; Lim et al., 2005), *OsSCR* transcripts accumulate in the epidermis and mark developing stomata (Kamiya et al., 2003). Notably, although duplicate SCR genes are now known to be present in both species, Kamiya et al. (2003) were only aware of one in rice. Given the high level of sequence similarity between the two sequences (CDS 96% sequence similarity), however, it is probable that the *in situ* hybridisation analysis detected transcripts of both genes (Kamiya et al., 2003). In maize, phenotypic perturbations are only observed in double *Zmscr1;Zmscr1h* mutants (Hughes et al., 2019), whereas in rice *Osscr1* single mutants reportedly showed defects (Wu et al., 2019). Specifically, *Osscr1* but not *Osscr2* mutants exhibited reduced stomatal density, and stomatal patterning in *Osscr1;Osscr2* double mutants was similar to that seen in *Osscr1* single mutants (Wu et al., 2019). Because SCR duplicated independently in maize and rice (Fig. 1), this observation raises the possibility that *OsSCR1* and *OsSCR2* have diverged in function, such that OsSCR1 patterns the epidermis and OsSCR2 patterns the inner leaf layers. This possibility needs further investigation to determine the extent to which SCR function has diverged both within and between rice and maize.

Here we show, contrary to previous reports, that *OsSCR1* and *OsSCR2* genes function redundantly in rice. Double but not single mutants exhibit severe growth and root patterning defects that are similar to those seen in maize and *Arabidopsis*. However, the rice double mutants do not show the inner leaf patterning defects observed in maize and instead leaves formed post-embryogenesis are almost entirely devoid of stomata, a phenotype far more severe than previously reported. Fittingly, the expression of known stomatal regulators were reduced in double mutants. No such reduction in stomata is found on the abaxial leaf surface in the equivalent maize mutant, although a minor reduction in stomatal density is found on the adaxial surface. Taken together, our results demonstrate that SCR has been recruited into two separate developmental pathways in two closely related monocot species that diverged around 50 million years ago (Vicentini et al., 2008; Wolfe et al., 1989).

## RESULTS

### Generation of *Osscr* mutant lines

**Fig. 2.**
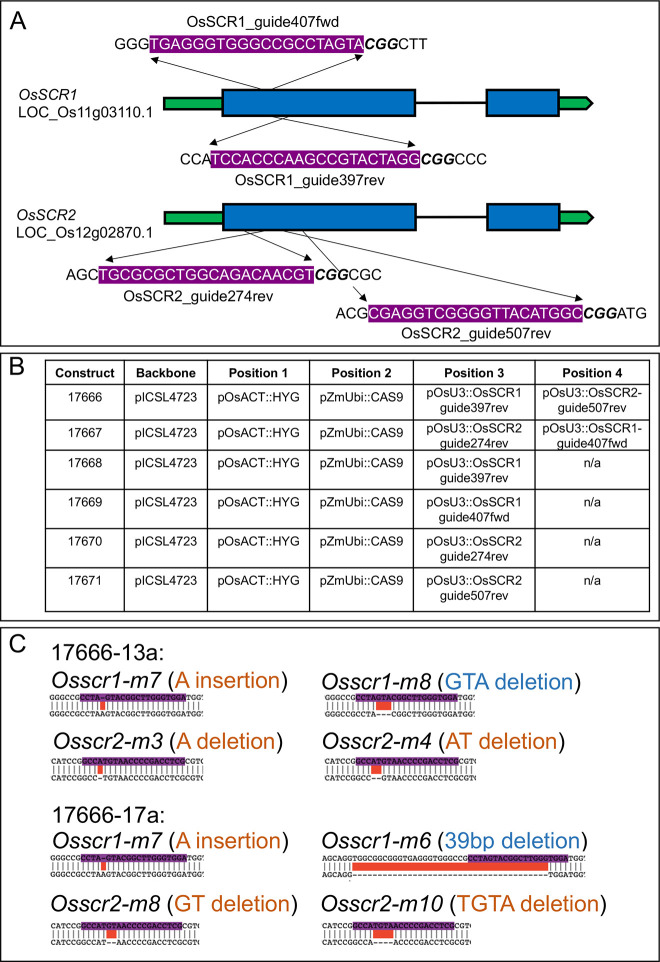
**Generation of CRISPR mutants.** (A) Cartoon depiction of guides designed for *OsSCR1* and *OsSCR2* genes. UTR regions are depicted in green, exons in blue and introns as single black lines. Guide sequences are written 5′→3′ and highlighted in purple above their approximate position either above (forward guides) or below (reverse guides) the gene model. (B) Details of each construct used in the study. (C) Summary of mutant alleles in two independent double mutants used for phenotypic characterisation. Mutations that lead to a frame-shift of the downstream protein are written in orange; mutations that do not alter the reading frame are written in blue. Sequences are wild type (top) and mutant allele (bottom).

To assess the role of SCR in rice development, four CRISPR guide RNAs (gRNA) were designed (two that targeted *OsSCR1* and two that targeted *OsSCR2*) and cloned into constructs that would enable all relevant combinations of knockout mutants to be generated and assessed (Fig. 2A,B; Fig. S1). All guides target regions near the 5′ end of the gene sequence such that out-of-frame deletions or insertions lead to complete loss of a functional protein (Fig. 2A). All four guides edited successfully and a number of mutant T0 plants were obtained. Mutant alleles were sequenced and those used for phenotypic characterisation are summarised in Fig. 2 and Fig. S1. In lines transformed with constructs designed to knock out both SCR genes, it was notable that no T0 plants were identified in which all four alleles were likely to encode non-functional proteins. Instead, all T0 plants screened were found to encode at least one in-frame deletion or insertion, and were thus predicted to have at least one functional allele. Given the known perturbed growth phenotypes associated with *scr* mutants in *Arabidopsis* and maize (Dhondt et al., 2010; Hughes et al., 2019), we hypothesised that complete loss-of-function mutants in rice may not regenerate from tissue culture and/or set seed. Therefore, edited but construct-free T1 plants were identified from two independent lines (both generated using the 17666 construct) in which one allele was predicted to be an in-frame deletion that would likely not substantially alter the protein sequence. As such, plants should grow normally and T1 progeny will segregate one in four for a full loss-of-function double mutant. Line 17666-13a contained the alleles *Osscr1-m7/m8;Osscr2-m3/m4* and line 17666-17a contained the alleles *Osscr1-m6/m7;Osscr2-m8/m10* (Fig. 2C). Because the two lines encoded non-identical alleles, we deduced that they were independently generated from tissue culture. These two lines, alongside equivalent single mutant lines (Fig. S1E), were prioritised for phenotypic analysis (Fig. 2C).

### *Osscr1;Osscr2* double mutants have perturbed growth and root development

**Fig. 3.**
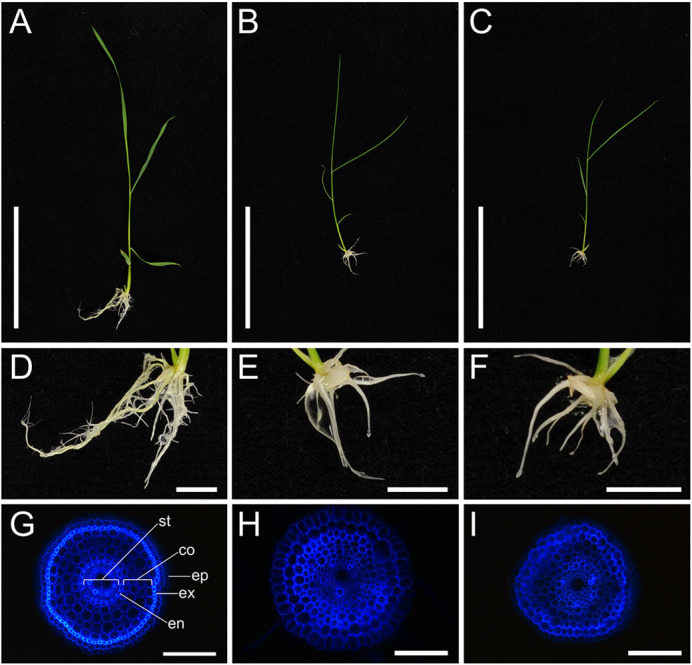
***Osscr1;Osscr2* mutants have perturbed shoot and root development.** (A-F) Photographs of wild-type (A,D), *Osscr1-m7;Osscr2-m3* (B,E) and *Osscr1-m7;Osscr2-m10* (C,F) plants 14 days after sowing. Scale bars: 10 cm in A-C; 1 cm in D-F. (G-I) Fresh cross-sections of kitaake wild-type (G), *Osscr1-m7;Osscr2-m3* (H) and *Osscr1-m7;Osscr2-m10* (I) roots, imaged under ultra-violet illumination. Scale bars: 100 µm. In G, the epidermis (ep), exodermis (ex), cortex (co), endodermis (en) and stele (st) are labelled.

Detailed phenotypic analysis was undertaken on the progeny of self-pollinated *Osscr1/+;Osscr2* plants from both independent lines (where + indicates an in-frame edit and thus predicted wild-type protein function). In each case, roughly one quarter of the progeny consistently exhibited a striking growth phenotype, whereby plants had very few roots and the shoots were very small with rolled up leaves (Fig. 3A-F). This phenotype was also observed in other T1 lines that were not taken forward for detailed analysis. In all cases, sequencing confirmed that these plants were homozygous loss-of-function double mutants. The two lines analysed in detail are referred to from here on as *Osscr1-m7;Osscr2-m3* (*1-m7;2-m3* for short) and *Osscr1-m7;Osscr2-m10* (*1-m7;2-m10* for short). Double mutants grew more slowly than wild type and rarely survived beyond 3-4 weeks after sowing. In contrast, both single mutants and plants with at least one in-frame deletion displayed normal growth phenotypes and appeared identical to wild type (Fig. S2), indicating that OsSCR1 and OsSCR2 function redundantly to regulate growth.

It has previously been demonstrated that despite differences in monocot and dicot root development, SCR has a conserved role in endodermal patterning in both maize and *Arabidopsis* (Hughes et al., 2019; Laurenzio et al., 1996; Lim et al., 2005). To establish whether this role is also conserved in rice, we analysed cross-sections of *Osscr1;Osscr2* roots. Because double mutants form shorter roots than wild type (Fig. 3A-F), we compared unbranched roots with similar diameters and obtained cross-sections from the maturation zone above the root tips (Fig. 3G-I). *Osscr1;Osscr2* roots displayed a severely perturbed phenotype. Specifically, cell layers were disorganised throughout, with no obvious endodermal layer separating the vasculature from the cortex, and no obvious exodermal layer separating the cortex from the epidermis (Fig. 3G-I). This phenotype strongly resembles that found in maize *Zmscr1;Zmscr1h* roots, indicating that the role of SCR in root patterning is conserved in rice, maize and *Arabidopsis*.

### *Osscr1;Osscr2* double mutants show no obvious inner leaf patterning defects

**Fig. 4.**
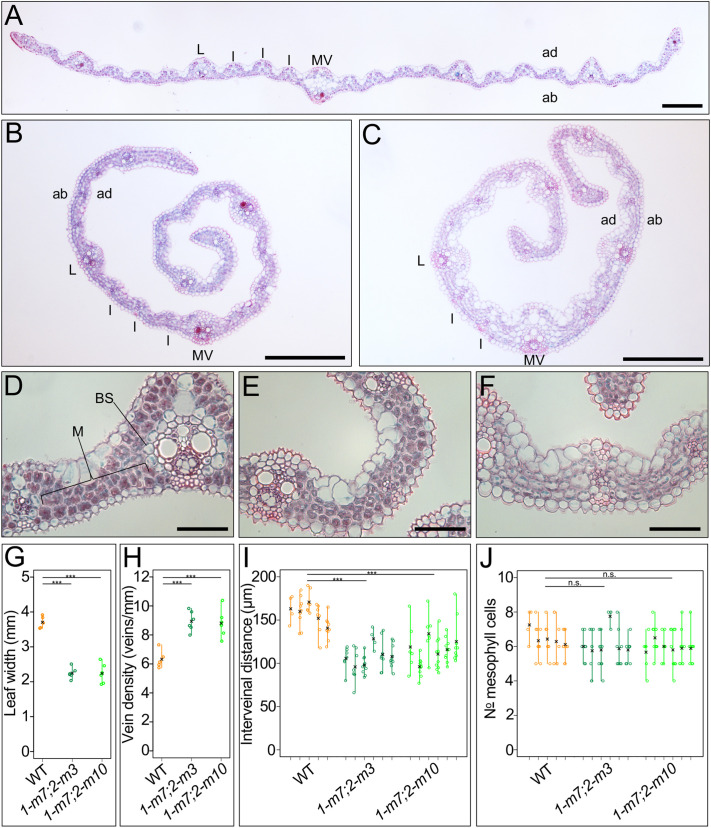
**Inner leaf cell patterning is unperturbed in *Osscr1;Osscr2* mutants.** (A-F) Cross-sections of wild-type (A,D), *Osscr1-m7;Osscr2-m3* (B,E) and *Osscr1-m7;Osscr2-m10* (C,F) leaves. Leaves were sampled from the mid-point of fully expanded leaf 5, 18 days after sowing. In A-C, the mid-vein (MV), examples of lateral (L) and intermediate (I) veins, and the abaxial (ab) and adaxial (ad) surfaces are indicated. In D, mesophyll (M) and bundle-sheath (BS) cells are indicated. Scale bars: 100 µm in A-C; 50 µm in D-F. (G-J) Quantification of leaf width (G), vein density (H), inter-veinal distance (I) and the number of mesophyll cells separating veins (J). The means for each plant are indicated by a black cross. In I and J, each line represents an individual leaf from a distinct plant; each circle represents one inter-veinal region. Samples sizes are wild type, *n*=5; *Osscr1-m7;Osscr2-m3*, *n*=6; *Osscr1-m7;Osscr2-m10*, *n*=6. Statistical significance between each genotype was assessed using one-way ANOVA and Tukey's HSD post-hoc tests: n.s., *P*>0.05; ***P≤0.001.

In maize, *Z*mSCR1 and ZmSCR1h function redundantly to regulate mesophyll specification and cell division in the inner leaf (Hughes et al., 2019). To assess whether this function is conserved in rice, which has many more mesophyll cells positioned between veins than maize, we examined cross-sections of *Osscr1;Osscr2* mutant leaves (Fig. 4A-F). Despite being significantly narrower (Fig. 4G) and more rolled (Fig. 4B,C), double mutant leaves did not exhibit any patterning defects in the inner leaf. Although vein density was significantly increased (Fig. 4H) and inter-veinal distance was significantly reduced (Fig. 4I), these effects are likely to be caused by altered leaf width and/or cell size, rather than by any direct cell-patterning defects. In support of this suggestion, traits that are characteristic of patterning defects in *Zmscr1;Zmscr1h* mutants were absent. That is, there was no reduction in the number of mesophyll cells separating veins (Fig. 4J), no ectopic bundle sheath or sclerenchyma cells and no fused vascular bundles (Fig. 4A-C). In summary, no evidence was found to support a role for *OsSCR1* or *OsSCR2* in inner leaf patterning in rice, indicating that, in this context, SCR function has diverged between rice and maize.

### OsSCR1 and OsSCR2 function redundantly to specify stomata

**Fig. 5.**
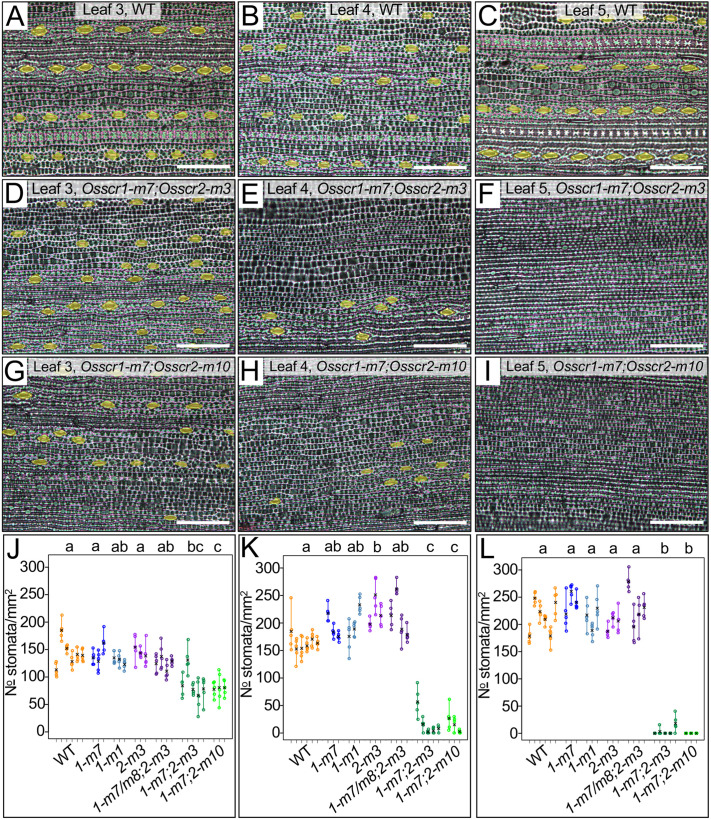
***Osscr1;Osscr2* leaves have almost no stomata.** (A-I) Abaxial impression of wild-type (A-C), *Osscr1-m7;Osscr2-m3* (D-F) and *Osscr1-m7;Osscr2-m10* (G-I) leaves 3 (A,D,G), 4 (B,E,H) and 5 (C,F,I). Stomata are false coloured yellow. Leaves were sampled at the mid-point along the proximal-distal axis either 14 (leaves 3 and 4) or 18 (leaf 5) days after sowing. Scale bars: 100 µm. (J-L) Quantification of stomatal density of leaf 3 (J), leaf 4 (K) and leaf 5 (L) for wild type (*n*=5), *Osscr1-m7* (*1-m7*) (*n*=3), *Osscr1-m1* (*1-m1*) (*n*=3), *Osscr2-m3* (*2-m3*) (*n*=3), *Osscr1-m7/m8;Osscr2-m3* (*1-m7/m8;2-m3*) (where *1-m8* is an in-frame deletion and thus functionally these are *Osscr2* single mutants) (*n*=4), *Osscr1-m7;Osscr2-m3* (*1-m7;2-m3*) (*n*=5) and *Osscr1-m7;Osscr2-m10* (*1-m7;2-m10*) (*n*=3). Different colours depict the different genotypes beneath each group. Each line represents an individual leaf from a distinct plant; each circle an image quantified for each leaf. The means for each plant are indicated by a black cross. Letters at the top of each plot indicate statistically different groups (*P*<0.05, one-way ANOVA and Tukey's HSD).

Given our finding that OsSCR1 and OsSCR2 function redundantly to regulate plant growth, we found the reported stomatal defects in *Osscr1* single mutants perplexing (Wu et al., 2019). We therefore sought to better understand the role of both genes in stomatal development (Fig. 5). In wild-type rice, three to four leaf primordia are initiated and at least partially patterned during embryogenesis (Itoh et al., 2005). To examine stomatal patterning in leaves formed both during embryogenesis and post-germination, we therefore quantified stomatal density on leaves 3, 4 and 5, using resin impressions of the leaf surface (Fig. 5A-I). Surprisingly, given previously published results, stomatal density was not reduced on the abaxial surface of any leaf in either *Osscr1* or *Osscr2* single mutants (Fig. 5J-L). In contrast, abaxial stomatal density was reduced to around 60% of wild-type in leaf 3 of double *Osscr1;Osscr2* mutants (Fig. 5A,D,G,J), and stomatal rows were not as clearly defined, with instances of clustering (Fig. 5D,G). These phenotypes were even more pronounced in leaves 4 and 5, with stomata rarely observed in leaf 4 (Fig. 5B,E,H,K) and never observed in leaf 5 of the *Osscr1-m7;Osscr2-m10* mutant (Fig. 5F,K). A few stomata were observed in leaf 5 of the *Osscr1-m7;Osscr2-m3* mutant, but only in two out of five individual plants examined (Fig. 5C,F,I,L). When stomata were present, they were in patches and in poorly defined rows (Fig. 5E,H). The adaxial surface of leaf 5 was also devoid of stomata in both double mutants (Fig. S3A-C). To confirm that these results were not a technical artefact of resin impressions, double mutant leaves were also examined directly under a scanning electron microscope. The images obtained confirmed the virtual absence of stomata on both surfaces of leaf 5 (Fig. S3D-G). No obvious aborted stomata or guard mother cells (GMCs) were seen in either double mutant, although assessing epidermal patterning of rice leaves is made challenging by the presence of wax and silica on the leaf surface (Fig. 5, Fig. S3). Notably, defects appear to be specifically associated with stomatal patterning because other epidermal cell types, such as bulliform cells, form in expected numbers and positions (Fig. S4). In summary, OsSCR1 and OsSCR2 redundantly regulate stomatal development, particularly in leaves that are initiated post-embryogenesis.

### OsSCR functions upstream of OsMUTE during stomatal development

**Fig. 6.**
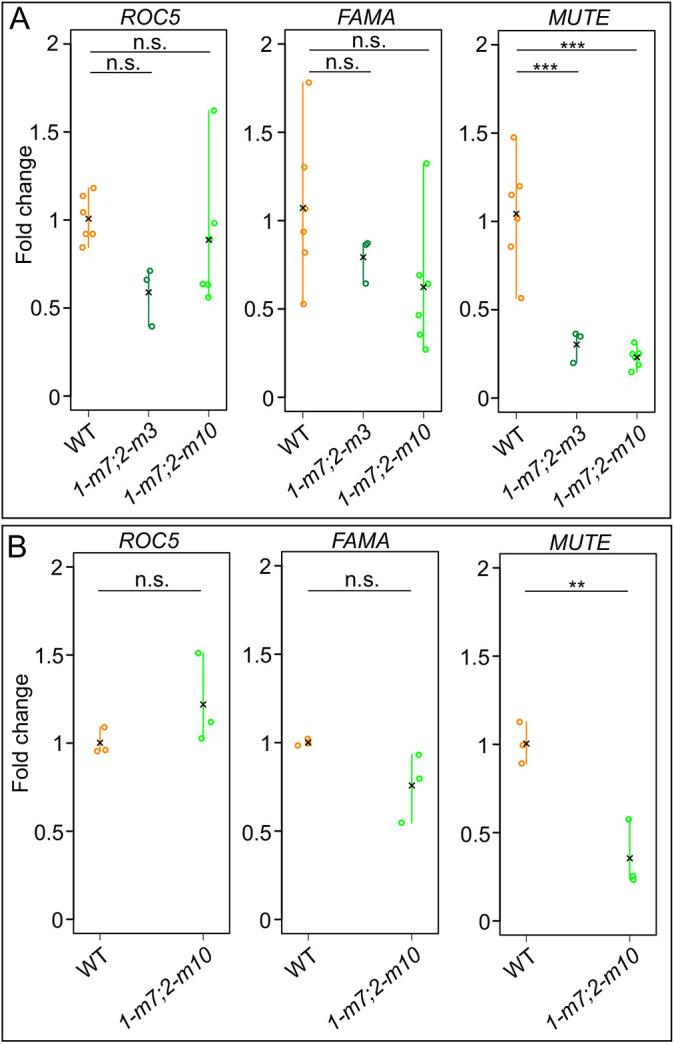
***OsMUTE* is strongly downregulated in *Osscr1;Osscr2* mutants.** (A,B) Quantitative RT-PCR analysis of *OsROC5, OsFAMA* and *OsMUTE* levels in wild-type, *Osscr1-m7;Osscr2-m3* (*1-m7;2-m3*) and *Osscr1-m7;Osscr2-m10* (*1-m7;2-m10*) plants. In A, both kitaake wild-type and mutant seedlings were harvested 6 days after sowing, whereas in B, kitaake wild type was harvested 4 days after sowing and compared with the same mutant samples in A. In each plot individual datapoints are biological replicates and means for each genotype are indicated by a black cross. Samples sizes in A are wild type, *n*=6; *Osscr1-m7;Osscr2-m3*, *n*=3; *Osscr1-m7;Osscr2-m10*, *n*=6. Samples sizes in B are wild type, *n*=3; *Osscr1-m7;Osscr2-m10*, *n*=3. Statistical significance between each genotype was assessed using one-way ANOVA and Tukey's HSD post-hoc tests: n.s., *P*>0.05; **P≤0.01; ***P≤0.001.

A number of genes that regulate stomatal specification and differentiation have been identified in monocots, largely due to having conserved roles with *Arabidopsis* orthologs. For example, in rice and Brachypodium, MUTE regulates GMC differentiation and subsidiary cell recruitment and FAMA regulates final stomatal patterning (Liu et al., 2009; Raissig et al., 2017; Wu et al., 2019). SPEECHLESS (SPCH) has also been implicated in controlling entry to the stomatal lineage in monocots (Raissig et al., 2016; Wu et al., 2019), but in line with previous studies in rice, we were unable to reliably detect SPCH expression at quantifiable levels (Liu et al., 2009). Therefore, to position SCR in this pathway we quantified *OsMUTE* and *OsFAMA* transcripts in developing *Osscr1;Osscr2* mutant leaves 6 days after sowing (Fig. 6). Given that the perturbed growth phenotype in *Osscr1;Osscr2* mutants may represent a developmental delay relative to wild type, we first quantified *ROC5* gene transcript levels. *ROC5* has been previously shown to mark the developing epidermis in rice (Ito et al., 2003)*.* Although *ROC5* levels appeared to be slightly reduced in the *Osscr1-m7;Osscr2-m3* line, this was not significant, and there was no consistent reduction in the *Osscr1-m7;Osscr2-m10* line (Fig. 6A). Therefore, we concluded that the relative amount of developing epidermal tissue is similar in both genotypes. In contrast, *OsMUTE* levels were drastically reduced in both *Osscr1;Osscr2* lines (Fig. 6A). There was a trend for reduced *OsFAMA* levels, but to a lesser extent and in a less consistent manner than *OsMUTE* (Fig. 6A). These results were confirmed using wild-type tissue harvested 4 days after sowing, when shoots were similarly sized to mutant seedlings harvested 6 days after sowing. In this comparison, *ROC5* levels were still not statistically different between wild type and mutant, but *OsMUTE* and *OsFAMA* transcript levels in the mutant showed the same pattern as seen in the previous comparison, with *OsMUTE* in particular strongly downregulated (Fig. 6B). Taken together, these data indicate that OsSCR1 and OsSCR2 function upstream of OsMUTE and OsFAMA in stomatal development, and thus that SCR may regulate entry into the stomatal specification pathway, particularly in non-embryonic leaves.

### Stomatal density is reduced only on the adaxial surface of *Zmscr1;Zmscr1h* mutants

**Fig. 7.**
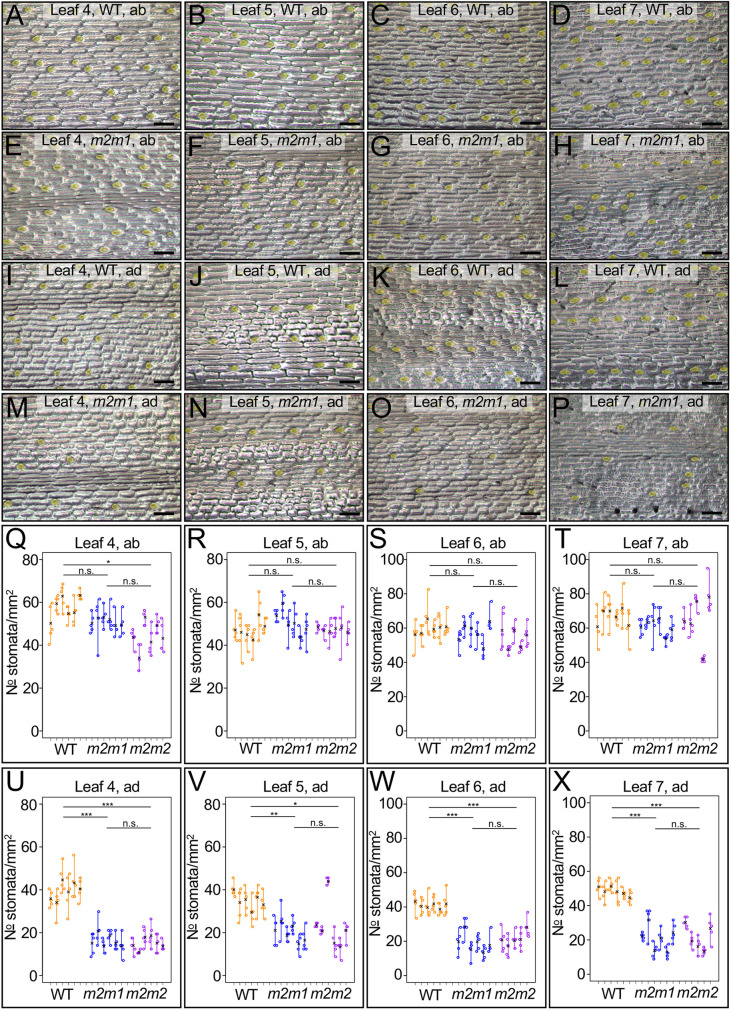
**Adaxial but not abaxial stomatal density is reduced in *Zmscr1;Zmscr1h* mutants.** (A-P) Epidermal impressions of abaxial (A-H) or adaxial (I-P) maize leaves from either segregating wild type (A-D,I-L) or *Zmscr1-m2;Zmscr1-m1* (*m2m1*) mutants (E-H,M-P), for leaves 4 (A,E,I,M), 5 (B,F,J,N), 6 (C,G,K,O) and 7 (D,H,L,P). Scale bars: 100 µm. Stomata are false coloured yellow. (Q-X) Stomatal density quantification for both abaxial (Q-T) and adaxial (U-X) leaf surfaces for leaves 4 (Q,U), 5 (R,V), 6 (S,W) and 7 (T,X). In each plot in Q-X, each line is an individual leaf from a distinct plant, and each datapoint is an image quantified for that leaf. Different colours depict the segregating *m2m1* wild-type line (orange), *m2m1* mutant (blue) and *m2m2* mutant (purple). Means for each plant are depicted by a black cross. Samples sizes are wild type, *n*=6; *m2m1*, *n*=6; *m2m2*, *n*=6 for leaves 4 and 5, and *n*=5 for leaves 6 and 7. Statistical significance between each genotype was assessed using one-way ANOVA and Tukey's HSD post-hoc tests: n.s., *P*>0.05; *P≤0.05; **P≤0.01; ***P≤0.001.

To assess whether a role in stomatal development is specific to rice or shared with other monocots, we assessed stomatal patterning in leaves 4 to 7 of two independent *Zmscr1;Zmscr1h* mutants (Fig. 7) (Hughes et al., 2019). Maize initiates up to five leaves during embryogenesis (Liu et al., 2013) and thus leaves 4 to 7 encompass the same developmental trajectory from embryonic to non-embryonic leaves as rice leaves 3 to 5. Whereas leaf 5 of *Osscr1;Osscr2* mutants formed very few stomata on either the abaxial or adaxial surface, leaf 7 of *Zmscr1;Zmscr1h* mutants showed no reduction in stomatal density on the abaxial surface (Fig. 7A-H,Q-T). There was a decrease in stomatal density on the adaxial surface (Fig. 7I-P,U-X); however, the reduction was not as great as seen in rice, and there was no difference between embryonic and non-embryonic leaves. Given that both *ZmSCR1* and *ZmSCR1h* are expressed in the inner layers of the maize leaf (Hughes et al., 2019), with no clear evidence for stomatal expression, the reduction in adaxial stomatal density in double mutants is likely an indirect consequence of loss of SCR function, although it is possible that some aspect of a stomatal patterning function is retained in maize. Intriguingly, ZmSCR1 has recently been demonstrated to move from the endodermis to the stele in maize roots, meaning that a non-cell-autonomous function in maize leaves cannot be ruled out (Ortiz-Ramírez et al., 2021). Whether an indirect or direct effect, however, the stomatal phenotype in maize double mutants is so distinct from that exhibited in rice double mutants that there is a high degree of divergence in SCR function between these species.

## DISCUSSION

It is increasingly appreciated that the same gene or regulatory module can be co-opted to regulate distinct developmental pathways both within and between species. In this study, we have demonstrated that, in rice, duplicate SCR genes redundantly regulate growth and root development in a manner that is shared with orthologous genes in *Arabidopsis* and maize (Fig. 3). In the context of leaf development, however, the rice genes have a divergent role in stomatal patterning. Gene function is necessary for stomatal development, with loss-of-function mutants virtually devoid of stomata on the leaf surface (Fig. 5). We position OsSCR1 and OsSCR2 upstream of the known stomatal regulators OsMUTE and OsFAMA (Fig. 6). The stomatal patterning perturbations in rice mutants are not exhibited in equivalent maize mutants (Fig. 7), and the inner leaf patterning perturbations described in maize mutants are not apparent in rice (Fig. 4). Taken together, these results demonstrate that, in the context of leaf development, SCR has distinct roles in two closely related monocot species.

Previous reports have suggested that OsSCR1 plays a more important role in stomatal development than OsSCR2 (Wu et al., 2019). Wu et al. (2019) found that stomatal density was reduced to around 50% that of wild type in leaf 5 of *Osscr1* single mutants, and that the *Osscr1;Osscr2* mutant phenotype was only slightly more severe. This stands in marked contrast to our results, where no decrease was seen in the single mutant but stomata were virtually absent in leaf 5 of the double mutant. It is not clear why these differences have arisen. Although an environmental contribution cannot be ruled out, our results clearly demonstrate that, contrary to previous reports, OsSCR1 and OsSCR2 function redundantly in stomatal development.

Our finding that the penetrance of the reduced stomatal density phenotype increases from leaf 3 to leaf 5 is intriguing. Rice normally forms three or four embryonic leaves (Itoh et al., 2005), and as such our phenotyping encompasses the transition from embryonic to non-embryonic leaf development. Little is known about whether distinct patterning mechanisms operate in embryonic and non-embryonic leaves. One possibility is that SCR plays a more crucial role in the stomatal development pathway in non-embryonic leaves, which could feasibly occur via altered expression levels. Alternatively, a closely related GRAS homolog that is expressed at higher levels during embryogenesis and functions partially redundantly with OsSCR1 and OsSCR2 might partially complement the phenotype in leaf 3. The obvious candidate for this homolog would be the rice ortholog of *Arabidopsis* SCL23, which has been found to function redundantly with SCR during root development (Long et al., 2015). However, the rice ortholog is not co-expressed with *OsSCR1* and *OsSCR2*, and is more highly expressed at P5, after stomatal patterning has occurred (van Campen et al., 2016). Thus, we hypothesize that OsSCR function in leaf development is inherently different in embryonic and non-embryonic contexts.

Given the virtual absence of stomata on leaves 4 and 5 of *Osscr1;Osscr2* mutants, it is counter-intuitive that *OsMUTE* is strongly downregulated, whereas the downstream gene *OsFAMA* is not. However, at the timepoint sampled (6 days after sowing), growth kinetic analyses show that under our growth conditions leaf 3 is normally at the plastochron (P) 4 stage, leaf 4 at P3 and leaf 5 at P2 (although there is some variability to this). *OsSCR1* and *OsSCR2* transcript levels are highest at P3, when initial stomatal patterning occurs, with expression maintained but decreased at P4, when stomatal differentiation occurs (van Campen et al., 2016). As such, because leaf 4 (which forms almost no stomata in the mutant) is at the stomatal patterning stage at the time of sampling, transcript levels of the patterning gene *OsMUTE* are reduced. In contrast, leaf 3 (which does form some stomata) is at the stomatal differentiation stage at the time of sampling. Thus transcript levels of the differentiation-associated gene *OsFAMA* are less affected than *OsMUTE* in the *Osscr1;Osscr2* mutant samples analysed.

The regulation of stomatal development in eudicots is well characterised (reviewed by Simmons and Bergmann, 2016), but it is only recently that equivalent regulators have been identified in monocots. Monocot grasses such as rice, maize and *Brachypodium* have complex stomata that develop subsidiary cells in addition to guard cell pairs (Stebbins and Shah, 1960). In *Brachypodium* and rice*,* stomatal cell files are initiated by a complex of three bHLH transcription factors [INDUCER OF CBF EXPRESSION 1 (ICE1), SPEECHLESS1 (SPCH1) and SPCH2] (Raissig et al., 2016; Wu et al., 2019). Within these cell files, asymmetric cell divisions lead to the formation of the GMCs. In *Brachypodium*, rice and maize, MUTE orthologs function both cell-autonomously to regulate the symmetrical division of the GMC to form guard cells and non-cell-autonomously to recruit subsidiary cells from adjacent cell files (Raissig et al., 2017; Wang et al., 2019; Wu et al., 2019). Our analysis supports a role for OsSCR1 and OsSCR2 upstream of MUTE because *OsMUTE* transcript levels were severely downregulated in *Osscr1;Osscr2* mutants. Given the absence of GMCs on leaf 5 of *Osscr1;Osscr2* mutants, the expression of *OsSCR* in developing stomata (Kamiya et al., 2003) and the role of SCR in promoting asymmetric cell divisions in *Arabidopsis* roots (Laurenzio et al., 1996), we hypothesise that, in rice (but not maize), SCR functions in stomatal cell files to regulate the asymmetric cell divisions that give rise to GMCs.

In rice, stomata are arranged in files flanking both sides of underlying veins and it has been hypothesised that a positional signal emanating from the veins acts to initiate development of those files (McKown and Bergmann, 2020; Nunes et al., 2020). In *Arabidopsis* roots, the SHORTROOT (SHR) protein acts as a positional signal, moving from the vasculature to the surrounding cell layer where it is bound by SCR. Activation of downstream targets by SHR/SCR then leads to an asymmetric cell division and the formation of the endodermis and cortex (Cui et al., 2007). The previous finding that SCR genes are expressed in developing stomata of rice leaves (Kamiya et al., 2003), and our validation of a redundant role for these genes in regulating stomatal development, suggests that, in rice, SHR may signal from the veins to the overlying epidermal regions. Signalling from the inner leaf to the epidermis has recently been demonstrated in *Arabidopsis*, where the light-responsive transcription factor HY5 induces STOMAGEN expression in the mesophyll, which then induces changes in stomatal patterning in the epidermis (Wang et al., 2021). Rice encodes two *SHR* orthologs, one of which is also expressed in developing stomata, albeit less obviously than seen for SCR (Kamiya et al., 2003). The second *OsSHR* gene is expressed at low levels in developing veins and when it is ectopically expressed in the bundle sheath cell layer surrounding the vein, additional stomatal files are initiated (Schuler et al., 2018). Given this finding, although there are several unresolved issues (such as how SHR can act as a vein-derived positional signal when one ortholog appears to be expressed in stomata), it is plausible that SCR acts cell-autonomously in stomatal cell files, interpreting a SHR signal from within the rice leaf to trigger the asymmetric cell divisions that generate GMCs.

In maize, *SCR* is expressed primarily in the inner leaf rather than in developing stomata (Hughes et al., 2019; Lim et al., 2005), and the phenotypes of the rice and maize mutants are highly diverged both in the inner leaf and the epidermis. Maize uses the C_4_ photosynthetic pathway, which is underpinned by Kranz anatomy, whereby vein density is higher than in species, such as rice, that use the ancestral C_3_ pathway. Notably, the higher vein density is not accompanied by higher stomatal density and, as such, some veins are not flanked by stomatal files. Given this disconnect, it is tempting to speculate that stomatal patterning in C_4_ monocots does not need to be as tightly integrated with venation patterning and thus that SCR is not necessary for stomatal development in maize. Of course, with only one C_3_ and one C_4_ monocot species characterised so far it is not possible to infer which function is ancestral, nor to assess whether the divergent functions reflect specific differences between maize and rice, or general differences between C_4_ and C_3_ species. If a role in stomatal patterning is linked to C_3_ leaf development, it is likely to be confined to the monocots because, in the C_3_ eudicot *Arabidopsis*, stomatal patterning is unaffected in *scr* mutants (Dhondt et al., 2010). Taken together, these results indicate that, in the context of leaf development, SCR function has been recruited into three largely distinct roles in maize, rice and *Arabidopsis*: patterning of mesophyll, stomatal and bundle-sheath cells, respectively.

## MATERIALS AND METHODS

### Plant growth

Seed of *Oryza sativa spp japonica* cv Kitaake (referred to as wild type throughout) were dehulled and sterilised by treatment with 70% ethanol (v/v) for 2 min followed by 25% sodium hypochlorite solution with a drop of Tween-20 for 15 min. Sterilised seed were rinsed five times with sterile deionised water and then placed on ½ MS media [2.15 g/l Murashige and Skoog salts and vitamins, 0.5 g/l MES, 4 g/l Phytagel (pH 5.8)] in an incubator (Panasonic, MLR-352) with 16* *h photoperiod and 30°C/25°C day-time/night-time temperature. After 7 days, seedlings were transferred to 50 ml Falcon tubes and watered with ¼ MS solution [1.07 g/l Murashige and Skoog salts and vitamins (pH 5.8)]. Falcon tubes were covered with clingfilm to maintain higher humidity until seedlings emerged from the tube. Phenotyping was undertaken on plants growing in Falcon tubes because *Osscr1;Osscr2* mutants did not survive beyond 3-4 weeks after sowing. Plants for seed propagation were transferred into 7.5 cm pots filled with clay granules (Profile, Porous Ceramic Topdressing and Construction Material) and placed in a controlled environment chamber with the same conditions as the incubator with light intensity 250-300μmol photons m^−2^ s^−1^. Trays of 15 plants were covered for around 1 week after transfer with a transparent bag to increase humidity. Plants were watered three times per week with a fertiliser solution [1.34 g/l Everris Peters Excel Cal-Mag Grower N.P.K. 15-5-15 (pH 5-6)], with 0.5 g/l chelated iron added to the solution on alternate weeks until flowering. Maize seedlings were grown as described previously (Hughes et al., 2019).

### CRISPR construct design and cloning

The rice SCR orthologs were obtained from a previously published phylogeny (Hughes et al., 2019). Sequences for each gene were obtained from phytozome V12 (https://phytozome-next.jgi.doe.gov) and guides targeting the first exon of each gene were designed using CRISPOR (Concordet and Haeussler, 2018). Guide oligonucleotides were synthesised with 4 bp golden gate sequences and Esp3I restriction sites added to facilitate cloning into a golden gate module. All golden gate reactions were carried out using a standard one-tube reaction as described previously (Engler et al., 2008). Complementary oligonucleotides were mixed in a 1:1 ratio and heated to 99°C before being left to cool for 1 h at room temperature and anneal. Annealed guides were diluted 200-fold for cloning into either module EC15768 (position 3, reverse) or EC15769 (position 4, reverse) (Fig. S1A). The resultant level 1 modules contained each guide in a full RNA scaffold sequence, driven by the OsU3 promoter (Fig. S1A). Promoter-guide modules were assembled into final level 2 transformation constructs in the pICSL4723 backbone with hygromycin resistance and CAS9 modules (Fig. S1A).

### Rice transformation and tissue culture

Constructs were transformed into *Agrobacterium tumefaciens* strain EHA105 and then used to transform Kitaake rice using a modified transformation protocol (Toki et al., 2006; Wang et al., 2017).

### Genotyping

Initial screening of T0 plants was undertaken with genomic DNA extracted using a modified sodium dodecyl sulfate (SDS) 96-well plate method (Hughes et al., 2019), whereby leaf tissue was frozen in liquid nitrogen and homogenised prior to the addition of extraction buffer. Hygromycin primers (Fig. S1B) were used to screen for successfully transformed T0 plants. Primers were designed to specifically amplify the first exon of either *OsSCR1* or *OsSCR2* (Fig. S1B), which worked efficiently when template genomic DNA was extracted using a previously published CTAB method (Hughes et al., 2019). The resultant amplicon was digested with a restriction enzyme predicted to cut at the intact guide site (Fig. S1C). If undigested (and thus edited), the amplicon was cloned into pJET (CloneJET, Thermofisher) and colonies sequenced by Sanger sequencing until both allele sequences were known. Seeds were collected from T0 plants of interest and T1 progeny grown to identify mutated plants that lacked the construct. This was achieved using the same hygromycin primers alongside a pair of primers amplifying the rice ubiquitin gene, to ensure that the failure to amplify hygromycin was not due to low DNA quality. Specific genotyping assays were designed to distinguish pairs of alleles in two independent T1 *Osscr1;Osscr2* mutants (Fig. S1D). All PCR reactions were undertaken with GoTaq DNA polymerase (Promega) with cycling conditions 95°C for 5 min; 35 cycles of 95°C for 30 s, 57-61°C for 30 s and 72°C for 60-90 s; and 72°C for 5 min. Betaine (1 M; Sigma Aldrich) was added to all amplifications of both SCR genes due to high-GC content. Restriction digestions were undertaken directly on the amplified PCR product, with 3µl of a 10µl PCR reaction used in a 10 µl digestion at the recommended digestion temperature overnight.

### Histology

Seminal roots were cut in the maturation zone of both Kitaake wild type and *Osscr1;Osscr2* mutants 15 days after sowing, and positioned in 3% agarose blocks. Roots were sectioned on a Leica VT1200S vibratome at 60 µm and the resultant sections floated on slides in deionised water. Sections were imaged using a Leica DMRB microscope and ultraviolet illumination with a DFC7000T camera and Leica LASX image analysis software.

Inner leaf phenotyping was undertaken on leaf segments cut at the mid-point of fully expanded leaf 5 at 18 days after sowing and fixed by submersion in 3:1 ethanol:acetic acid for 30 min followed by storage in 70% ethanol. Leaf tissue was wax infiltrated using a Tissue-Tek VIP machine (Sakura, www.sakura.eu) using the protocol published previously (Hughes et al., 2019), and the resultant wax blocks sectioned at 10 µm. Sections were cleared using Histoclear (10 min, ×2), followed by submersion in 100% and 70% ethanol (twice each for 2 min). Sections were stained using safranin O (1% in 50% ethanol) for 90 min, rinsed in 70% ethanol (twice for 2 min) and counterstained with Fast Green (0.03% in 95% ethanol) for 3 s per slide. Finally, slides were rinsed in 100% ethanol (twice for 2 min), then 100% histoclear (5 min) and mounted using a drop of DPX mounting medium. Images were obtained using bright-field illumination on the same microscope and software described above.

Stomatal impressions were taken using dental resin (Perfection Plus, Impress Plus Medium Body Fast Set) applied to the mid-point of leaves 3, 4 and 5 on either the abaxial or adaxial surface. Once applied, resin was left to dry for 5 min and then the leaf removed. Clear nail varnish (Rimmel) was applied to the dental resin impressions and left to dry for at least 5 min, before being peeled off and floated on deionised water, which was blotted off to dry impressions to the slide. Sections were imaged using phase-contrast illumination on the same microscope described above. For quantification, five 10× images were taken from random parts of each peel, and a higher magnification 20× image taken for presentation. Stomata were counted and divided by the area of peel to calculate stomatal density. For scanning electron microscopy rice leaves were attached directly to a stub without any pre-treatment and imaged directly on a scanning electron microscope (JEOL JSM5510, 15kV).

### Quantitative RT-PCR analysis

Wild-type and mutant seed were sterilised and plated on ½ MS media as described above. After either 4 (wild type) or 6 (wild type and mutant) days, whole shoots from each plant were removed and the length of each measured before freezing in liquid nitrogen. Total RNA was extracted using the Qiagen RNeasy kit and DNA contamination removed using Turbo DNase. 2 µg of RNA was used for cDNA synthesis and cDNA quality checked with ubiquitin primers that amplify distinct products from genomic DNA and cDNA.

Primers for two housekeeping genes (OsACTIN and OsUBQ5) were obtained from previously published work (Jain et al., 2006; Wang et al., 2017), and primers for *OsMUTE* (LOC_Os05g51820), *OsFAMA* (LOC_Os05g50900) and *OsROC5* (LOC_Os02g45250) were designed in the CDS of each gene using Primer3Plus (Fig. S5A). Quantitative RT-PCR analysis was undertaken using SYBR-green with cycle conditions 95°C for 10 min, then 40 cycles of 95°C for 15 s and 60°C for 1 min. Melt curves were obtained between 60°C and 95°C to establish that a single product was amplified for each primer pair (Fig. S5B). RT-PCR was undertaken to confirm primers amplified an amplicon of the correct size, and primer efficiency confirmed to be >80% using the qPCR miner algorithm from a test qRT-PCR run (Zhao and Fernald, 2005). Three technical replicates were obtained for each sample and confirmed to have Ct values with a range of less than ∼0.5 once outliers were removed. All comparisons were run on the same plate alongside water controls, and as such Kitaake wild-type samples were repeated alongside both independent mutant backgrounds. Ct values were calculated using the qPCR miner algorithm (Zhao and Fernald, 2005) and fold-change values using the 2−^ΔΔCT^ method (Livak and Schmittgen, 2001). The overall average wild-type across all samples was used to compare each individual wild type to indicate the range of the wild-type data. Mutant samples were then compared with the same overall wild-type average and, as such, values of less than 1 indicate a relative reduction compared with wild type.

## Supplementary Material

Supplementary information

Reviewer comments

## References

[DEV200410C1] Concordet, J.-P. and Haeussler, M. (2018). CRISPOR: intuitive guide selection for CRISPR/Cas9 genome editing experiments and screens. *Nucleic Acids Res.* 46, W242-W245. 10.1093/nar/gky354 29762716 PMC6030908

[DEV200410C2] Conklin, P. A., Strable, J., Li, S. and Scanlon, M. J. (2019). On the mechanisms of development in monocot and eudicot leaves. *New Phytol.* 221, 706-724. 10.1111/nph.15371 30106472

[DEV200410C3] Cui, H., Levesque, M. P., Vernoux, T., Jung, J. W., Paquette, A. J., Gallagher, K. L., Wang, J. Y., Blilou, I., Scheres, B. and Benfey, P. N. (2007). An evolutionarily conserved mechanism delimiting SHR movement defines a single layer of endodermis in plants. *Science (New York, N.Y.)* 316, 421-425. 10.1126/science.1139531 17446396

[DEV200410C4] Cui, H., Kong, D., Liu, X. and Hao, Y. (2014). SCARECROW, SCR-LIKE 23 and SHORT-ROOT control bundle sheath cell fate and function in Arabidopsis thaliana. *Plant J.* 78, 319-327. 10.1111/tpj.12470 24517883

[DEV200410C5] Dhondt, S., Coppens, F., de Winter, F., Swarup, K., Merks, R. M. H., Inze, D., Bennett, M. J. and Beemster, G. T. S. (2010). SHORT-ROOT and SCARECROW regulate leaf growth in arabidopsis by stimulating s-phase progression of the cell cycle. *Plant Physiol.* 154, 1183-1195. 10.1104/pp.110.158857 20739610 PMC2971598

[DEV200410C6] Dolan, L., Janmaat, K., Willemsen, V., Linstead, P., Poethig, S., Roberts, K. and Scheres, B. (1993). Cellular organisation of the Arabidopsis thaliana root. *Development (Cambridge, England)* 119, 71-84. 10.1242/dev.119.1.71 8275865

[DEV200410C7] Engler, C., Kandzia, R. and Marillonnet, S. (2008). A one pot, one step, precision cloning method with high throughput capability. *PLoS ONE* 3, e3647. 10.1371/journal.pone.0003647 18985154 PMC2574415

[DEV200410C8] Guo, X., Wang, L. and Dong, J. (2021). Establishing asymmetry: stomatal division and differentiation in plants. *New Phytol.* 232, 60-67. 10.1111/nph.17613 34254322 PMC8429090

[DEV200410C9] Hughes, T. E. and Langdale, J. A. (2020). SCARECROW gene function is required for photosynthetic development in maize. *Plant Direct* 4, e00264. 10.1002/pld3.264 32999956 PMC7507539

[DEV200410C10] Hughes, T. E., Sedelnikova, O. V., Wu, H., Becraft, P. W. and Langdale, J. A. (2019). Redundant SCARECROW genes pattern distinct cell layers in roots and leaves of maize. *Development (Cambridge, England)* 146, dev177543. 10.1242/dev.177543 PMC6679360 31235633

[DEV200410C11] Ito, M., Sentoku, N., Nishimura, A., Hong, S.-K., Sato, Y. and Matsuoka, M. (2003). Roles of rice GL2-type homeobox genes in epidermis differentiation. *Breed. Sci.* 53, 245-253. 10.1270/jsbbs.53.245

[DEV200410C12] Itoh, J.-I., Nonomura, K.-I., Ikeda, K., Yamaki, S., Inukai, Y., Yamagishi, H., Kitano, H. and Nagato, Y. (2005). Rice plant development: from zygote to spikelet. *Plant Cell Physiol.* 46, 23-47. 10.1093/pcp/pci501 15659435

[DEV200410C13] Jain, M., Nijhawan, A., Tyagi, A. K. and Khurana, J. P. (2006). Validation of housekeeping genes as internal control for studying gene expression in rice by quantitative real-time PCR. *Biochem. Biophys. Res. Commun.* 345, 646-651. 10.1016/j.bbrc.2006.04.140 16690022

[DEV200410C14] Kamiya, N., Itoh, J. I., Morikami, A., Nagato, Y. and Matsuoka, M. (2003). The SCARECROW gene's role in asymmetric cell divisions in rice plants. *Plant J.* 36, 45-54. 10.1046/j.1365-313X.2003.01856.x 12974810

[DEV200410C15] Laurenzio, L. D., Wysocka-Diller, J. and Malamy, J. (1996). The SCARECROW gene regulates an asymmetric cell division that is essential for generating the radial organization of the arabidopsis root. *Cell* 86, 423-433. 10.1016/S0092-8674(00)80115-4 8756724

[DEV200410C16] Lim, J., Jung, J. W., Lim, C. E., Lee, M.-H., Kim, B. J., Kim, M., Bruce, W. B. and Benfey, P. N. (2005). Conservation and diversification of SCARECROW in maize. *Plant Mol. Biol.* 59, 619-630. 10.1007/s11103-005-0578-y 16244911 PMC1475827

[DEV200410C17] Liu, T., Ohashi-Ito, K. and Bergmann, D. (2009). Orthologs of Arabidopsis thaliana stomatal bHLH genes and regulation of stomatal development in grasses. *Development (Cambridge, England)* 136, 2265-2276. 10.1242/dev.032938 19502487

[DEV200410C18] Liu, W.-Y., Chang, Y.-M., Chen, S. C.-C., Lu, C.-H., Wu, Y.-H., Lu, M.-Y. J., Chen, D.-R., Shih, A. C.-C., Sheue, C.-R., Huang, H.-C. et al. (2013). Anatomical and transcriptional dynamics of maize embryonic leaves during seed germination. *Proc. Natl Acad. Sci. USA* 110, 3979-3984. 10.1073/pnas.1301009110 23431200 PMC3593892

[DEV200410C38] Livak, K. J. and Schmittgen, T. D. (2001). Analysis of relative gene expression data using real-time quantitative PCR and the 2(-Delta Delta C(T)) Method. *Methods* 25, 402-408. 10.1006/meth.2001.1262 11846609

[DEV200410C19] Long, Y., Goedhart, J., Schneijderberg, M., Terpstra, I., Shimotohno, A., Bouchet, B. P., Akhmanova, A., Gadella, T. W., Jr, Heidstra, R., Scheres, B. et al. (2015). SCARECROW-LIKE23 and SCARECROW jointly specify endodermal cell fate but distinctly control SHORT-ROOT movement. *Plant J.* 84, 773-784. 10.1111/tpj.13038 26415082

[DEV200410C20] McKown, K. H. and Bergmann, D. C. (2020). Stomatal development in the grasses: lessons from models and crops (and crop models). *New Phytol.* 227, 1636-1648. 10.1111/nph.16450 31985072

[DEV200410C21] Nunes, T. D. G., Zhang, D. and Raissig, M. T. (2020). Form, development and function of grass stomata. *Plant J.* 101, 780-799. 10.1111/tpj.14552 31571301

[DEV200410C22] Ortiz-Ramírez, C., Guillotin, B., Xu, X., Rahni, R., Zhang, S., Yan, Z., Coqueiro Dias Araujo, P., Demesa-Arevalo, E., Lee, L., Van Eck, J. et al. (2021). Ground tissue circuitry regulates organ complexity in maize and Setaria. *Science (New York, N.Y.)* 374, 1247-1252. 10.1126/science.abj2327 PMC8719420 34855479

[DEV200410C23] Raissig, M. T., Abrash, E., Bettadapur, A., Vogel, J. P. and Bergmann, D. C. (2016). Grasses use an alternatively wired bHLH transcription factor network to establish stomatal identity. *Proc. Natl Acad. Sci. USA* 113, 8326-8331. 10.1073/pnas.1606728113 27382177 PMC4961163

[DEV200410C24] Raissig, M. T., Matos, J. L., Gil, M. X. A., Kornfeld, A., Bettadapur, A., Abrash, E., Allison, H. R., Badgley, G., Vogel, J. P., Berry, J. A. et al. (2017). Mobile MUTE specifies subsidiary cells to build physiologically improved grass stomata. *Science* 355, 1215-1218. 10.1126/science.aal3254 28302860

[DEV200410C25] Schuler, M. L., Sedelnikova, O. V., Walker, B. J., Westhoff, P. and Langdale, J. A. (2018). SHORTROOT-mediated increase in stomatal density has no impact on photosynthetic efficiency. *Plant Physiol.* 176, 757-772. 10.1104/pp.17.01005 29127261 PMC5761779

[DEV200410C26] Simmons, A. R. and Bergmann, D. C. (2016). Transcriptional control of cell fate in the stomatal lineage. *Curr. Opin. Plant Biol.* 29, 1-8. 10.1016/j.pbi.2015.09.008 26550955 PMC4753106

[DEV200410C27] Stebbins, G. L. and Shah, S. S. (1960). Developmental studies of cell differentiation in the epidermis of monocotyledons: II. Cytological features of stomatal development in the Gramineae. *Dev. Biol.* 2, 477-500. 10.1016/0012-1606(60)90050-6 13862290

[DEV200410C28] Toki, S., Hara, N., Ono, K., Onodera, H., Tagiri, A., Oka, S. and Tanaka, H. (2006). Early infection of scutellum tissue with Agrobacterium allows high-speed transformation of rice. *Plant J.* 47, 969-976. 10.1111/j.1365-313X.2006.02836.x 16961734

[DEV200410C29] van Campen, J. C., Yaapar, M. N., Narawatthana, S., Lehmeier, C., Wanchana, S., Thakur, V., Chater, C., Kelly, S., Rolfe, S. A., Quick, W. P. et al. (2016). Combined Chlorophyll fluorescence and transcriptomic analysis identifies the P3/P4 transition as a key stage in rice leaf photosynthetic development. *Plant Physiol.* 170, 1655-1674. 10.1104/pp.15.01624 26813793 PMC4775128

[DEV200410C30] Vicentini, A., Barber, J. C., Aliscioni, S. S., Giussani, L. M. and Kellogg, E. A. (2008). The age of the grasses and clusters of origins of C4 photosynthesis. *Glob. Change Biol.* 14, 2963-2977. 10.1111/j.1365-2486.2008.01688.x

[DEV200410C31] Wang, P., Khoshravesh, R., Karki, S., Tapia, R., Balahadia, C. P., Bandyopadhyay, A., Quick, W. P., Furbank, R., Sage, T. L. and Langdale, J. A. (2017). Re-creation of a key step in the evolutionary switch from C3 to C4 leaf anatomy. *Curr. Biol.* 27, 3278-3287.e6. 10.1016/j.cub.2017.09.040 29056456 PMC5678070

[DEV200410C32] Wang, H., Guo, S., Qiao, X., Guo, J., Li, Z., Zhou, Y., Bai, S., Gao, Z., Wang, D., Wang, P. et al. (2019). BZU2/ZmMUTE controls symmetrical division of guard mother cell and specifies neighbor cell fate in maize. *PLoS Genet.* 15, e1008377. 10.1371/journal.pgen.1008377 31465456 PMC6738654

[DEV200410C33] Wang, S., Zhou, Z., Rahiman, R., Lee, G. S. Y., Yeo, Y. K., Yang, X. and Lau, O. S. (2021). Light regulates stomatal development by modulating paracrine signaling from inner tissues. *Nat. Commun.* 12, 1-13. 10.1038/s41467-021-23728-2 34099707 PMC8184810

[DEV200410C34] Wolfe, K. H., Gouy, M., Yang, Y. W., Sharp, P. M. and Li, W. H. (1989). Date of the monocot-dicot divergence estimated from chloroplast DNA sequence data. *Proc. Natl Acad. Sci. USA* 86, 6201-6205. 10.1073/pnas.86.16.6201 2762323 PMC297805

[DEV200410C35] Wu, Z., Chen, L., Yu, Q., Zhou, W., Gou, X., Li, J. and Hou, S. (2019). Multiple transcriptional factors control stomata development in rice. *New Phytol.* 223, 220-232. 10.1111/nph.15766 30825332

[DEV200410C36] Wysocka-Diller, J. W., Helariutta, Y., Fukaki, H., Malamy, J. E. and Benfey, P. N. (2000). Molecular analysis of SCARECROW function reveals a radial patterning mechanism common to root and shoot. *Development (Cambridge, England)* 127, 595-603. 10.1242/dev.127.3.595 10631180

[DEV200410C37] Zhao, S. and Fernald, R. D. (2005). Comprehensive algorithm for quantitative real-time polymerase chain reaction. *J. Comput. Biol.* 12, 1047-1064. 10.1089/cmb.2005.12.1047 16241897 PMC2716216

