# Supplementary material for: SCARECROW is deployed in distinct contexts during rice and maize leaf development
Source: Development. 2022 Mar 30;149(7):dev200410. doi: 10.1242/dev.200410 (PMC8995083; doi:10.1242/dev.200410)
Supplement: Supplementary information [file develop-149-200410-s1.pdf]

A

L1 construct	Backbone	PU	SC/guide	T
17100	pL1V-R1-47802	pL0M-PU-pOsAct1-intron1-15216	pL0M-SC-HYG-15069	pL0M-T-Nos-1-41421
17738	pL1V-R2-47811	pL0M-PU-pZmUbi-intron1-15455	pL0M_SC_Cas9p-17570	pL0M-T-Nos-1-41421
17795	pL1V-R3-sgRNA pOsU3 scaffold-15768	n/a	OsSCR1-g397	n/a
17796	pL1V-R3-sgRNA pOsU3 scaffold-15768	n/a	OsSCR1-g407	n/a
17797	pL1V-R3-sgRNA pOsU3 scaffold-15768	n/a	OsSCR2-274	n/a
17798	pL1V-R3-sgRNA pOsU3 scaffold-15768	n/a	OsSCR2-507	n/a
33101	pL1V-R4-sgRNA pOsU3 scaffold-15769	n/a	OsSCR1-g407	n/a
33102	pL1V-R4-sgRNA pOsU3 scaffold-15769	n/a	OsSCR2-507	n/a

L2 construct	Backbone	Position 1	Position 2	Position 3	Position 4	End-linker
EC17666	pICSL-4723	pL1M-R1-pOsAct1-HYG-tNOS-17100	pL1M-R2-pZmUbi-Cas9p-tNOS-17738	pL1M-R3-pOsU3-CRISPR_OsSCR1-g397-17795	pL1M-R4-pOsU3-CRISPR_OsSCR2-g507-33102	pL1M-ELB4-49283
EC17667	pICSL-4723	pL1M-R1-pOsAct1-HYG-tNOS-17100	pL1M-R2-pZmUbi-Cas9p-tNOS-17738	pL1M-R3-pOsU3-CRISPR_OsSCR2-g274-17797	pL1M-R4-pOsU3-CRISPR_OsSCR1-g407-33101	pL1M-ELB4-49283
EC17668	pICSL-4723	pL1M-R1-pOsAct1-HYG-tNOS-17100	pL1M-R2-pZmUbi-Cas9p-tNOS-17738	pL1M-R3-pOsU3-CRISPR_OsSCR1-g397-17795	n/a	pL1M-ELB3-49277
EC17669	pICSL-4723	pL1M-R1-pOsAct1-HYG-tNOS-17100	pL1M-R2-pZmUbi-Cas9p-tNOS-17738	pL1M-R3-pOsU3-CRISPR_OsSCR1-g407-17796	n/a	pL1M-ELB3-49277
EC17670	pICSL-4723	pL1M-R1-pOsAct1-HYG-tNOS-17100	pL1M-R2-pZmUbi-Cas9p-tNOS-17738	pL1M-R3-pOsU3-CRISPR_OsSCR2-g274-17797	n/a	pL1M-ELB3-49277
EC17671	pICSL-4723	pL1M-R1-pOsAct1-HYG-tNOS-17100	pL1M-R2-pZmUbi-Cas9p-tNOS-17738	pL1M-R3-pOsU3-CRISPR_OsSCR2-g507-17798	n/a	pL1M-ELB3-49277

B

Primer name	Sequence (5'-3')
Hyg-F	CAACCAAGCTCTGATAGAGT
Hyg-R	GAAGAATCTCGTGCTTTCA
OsUbi-3F	GGGCGCCAAGATGCAAATCT
OsUbi-3R	GCAGTTGACAGCCCTAGGGT
OsSCR1ab-F1	TCTTGGTCTCCTCCTCTCGG
OsSCR1a_intR1	CCTTAATCATCCTATGGACTATGGTG
OsSCR1b_intR3	CTAGGTCACAATGTCAATGAGAAG

Primer name	Sequence (5'-3')
OsSCR1a_F1b	ACCACACGGACATGGACCT
OsSCR1a_R1	AGGTCGGGGTTACAAGGT
OsSCR1b_dCAPsm10_F	AACGTGCGTGAGATCATCCGGTCA
OsSCR1b_dCAPsm10_R	GACCGACGTGGGTGGTGG
OsSCR1b_F1	ACGGCTTGGGTGGATGGC
OsSCR1b_R1	GGGTCGGAGTTGAGGAGAGA

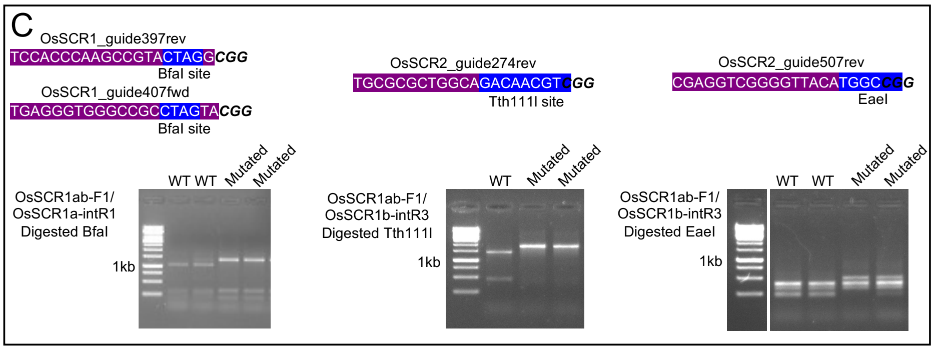

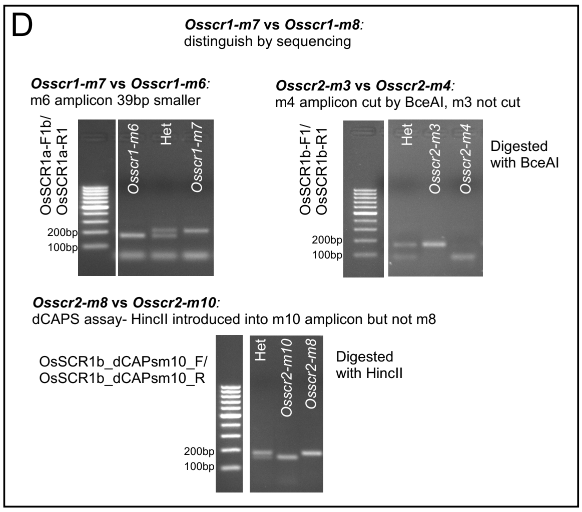

E

Allele name	Allele details	Resultant protein
Osscr1-m1	'A' deletion	Out-of-frame
Osscr1-m6	39bp deletion	In-frame
Osscr1-m7	'A' insertion	Out-of-frame
Osscr1-m8	'GTA' deletion	In-frame
Osscr2-m3	'A' deletion	Out-of-frame
Osscr2-m4	'AT' deletion	Out-of-frame
Osscr2-m8	'GT' deletion	Out-of-frame
Osscr2-m10	'TGTA' deletion	Out-of-frame

Fig. S1. Generation of CRISPR mutants. A) Golden gate modules and cloning used in this study. **B)** Genotyping primers used in this study. **C)** Restriction digest assays used to identify mutant alleles for each CRISPR guide. Guide sequence is depicted with purple highlighting and white lettering, the protospacer adjacent motif (PAM) sequence is depicted in black italic text, and the restriction enzyme site is depicted with blue highlighting. **D)** Distinguishing pairs of known alleles identified in Fig. 2C using different restriction enzyme assays. **E)** Summary of all alleles used for phenotyping in this study.

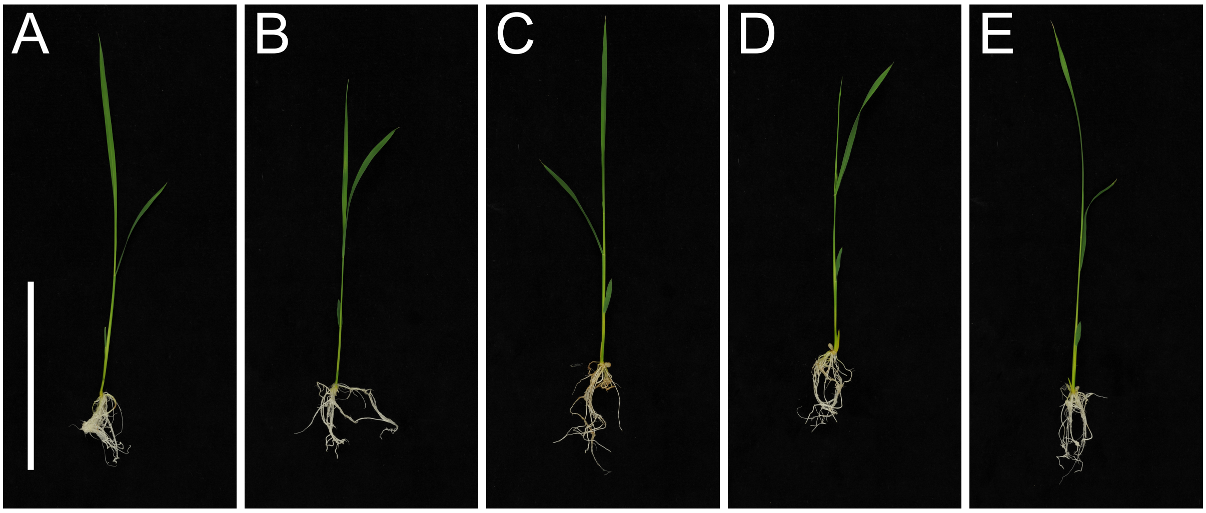

Fig. S2. *Osscr1* and *Osscr2* single mutants grow normally. A-E) Photographs of WT (A), *Osscr1-m7* (B), *Osscr1-m1* (C), *Osscr2-m3* (D) and *Osscr1-m8;Osscr2-m3* (E) plants taken 13 days after sowing. Orange alleles indicate out-of-frame mutations whereas blue alleles indicate an in-frame mutation not predicted to alter protein function. All mutants were obtained from independent lines or constructs, as indicated underneath the allele names. Scalebar in A is 10 cm and all images are at the same magnification.

Development • Supplementary information

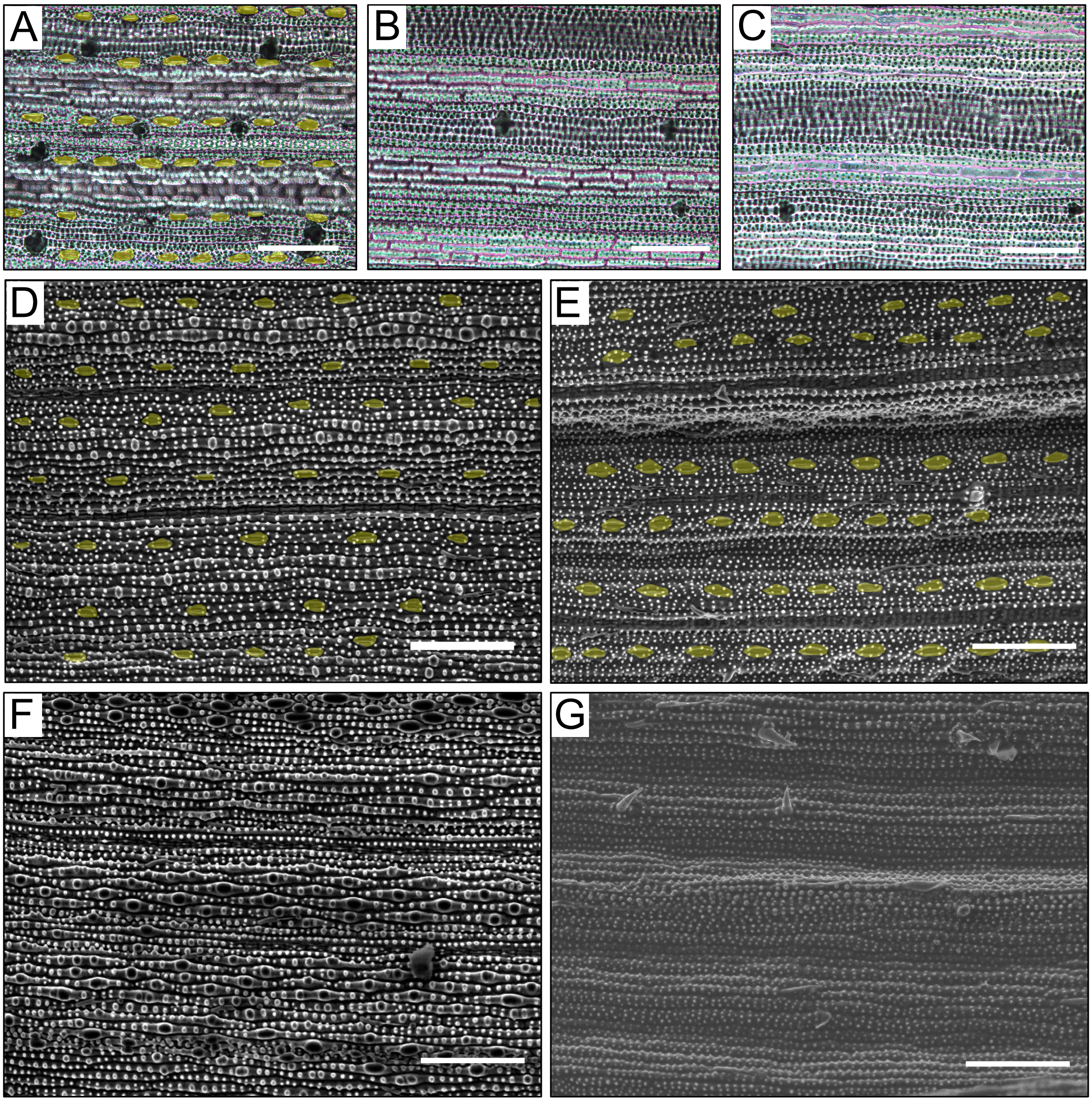

Fig. S3. Stomatal phenotypes are consistent on both sides of the leaf and not an artefact caused by taking impressions. A-C) Adaxial impressions of WT (A), *Osscr1-m7;Osscr2-m3* (B) and *Osscr1-m7;Osscr2-m10* (C) leaf 5. Scalebars are 100µm. **D-G)** Scanning electron microscope images of leaf 5 for kitaake WT (D and E) or *Osscr1-m7;Osscr2-m3* (F and G) abaxial (D and F) and adaxial (E and G) surfaces. Scalebars are 100µm. Stomata are false coloured yellow.

Development • Supplementary information

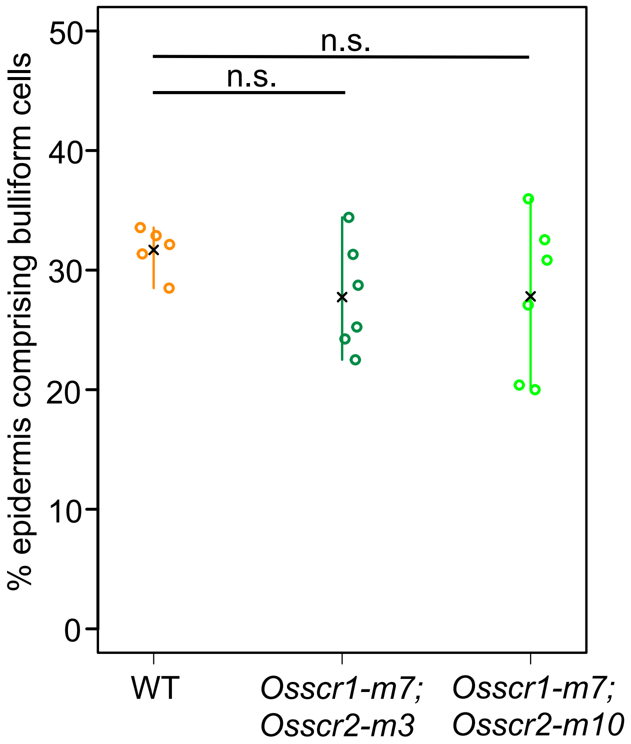

Fig. S4. % of the epidermis comprising bulliform cells is unchanged in *Osscr1;Osscr2* mutants. Quantification of the number of bulliform cells on the adaxial surface of fully expanded leaf 5 expressed as a percentage of the total number of epidermal cells. Each datapoint is a biological replicate, and the mean values for each genotype are indicated by a black cross. Samples sizes are WT, n=5; *Osscr1-m7;Osscr2-m3*, n=6; *Osscr1-m7;Osscr2-m10*, n=6. Statistical significance between each genotype was assessed using one-way ANOVA: n.s. P>0.05.

A

Gene	Accession/ ref	Forward primer (5'-3')	Reverse primer (5'-3')	Product size (bp)
OsACTIN	Wang *et al.* (2017)	GGCACCACACCTTCTACAAT	CTCACACCATCACCAGAGT	223
OsUBQ5	Jain *et al.* (2006)	ACCACTTCGACCGCCACTACT	ACGCCTAAGCCTGCTGGTT	69
OsROC5	LOC_Os02g45250	ATACCCAGCACCAGATCCAA	CATCTGTGTGCGCTTGTTCT	146
OsMUTE	LOC_Os05g51820	TCAACATCACCACCATGGAC	CATGAAGGACTGGTGGACCT	113
OsFAMA	LOC_Os05g50900	GAGATGGCGGAGAACAAGTC	CATGTGCATCTCCTCCAGTG	138

B

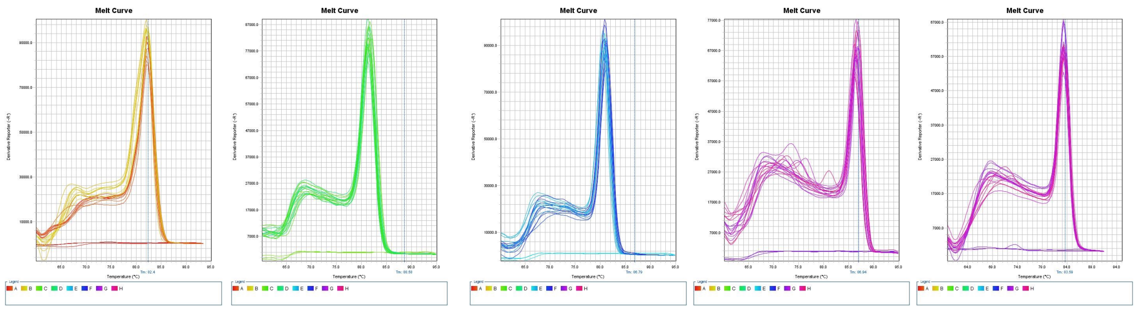

Fig. S5. Quantitative RT-PCR primer design and validation. A) Primer sequences and product sizes. **B)** Melt-curves for each primer pair used in the study.

Development • Supplementary information
