# Supplementary material for: SCARECROW is deployed in distinct contexts during rice and maize leaf development
Source: Development. 2022 Mar 30;149(7):dev200410. doi: 10.1242/dev.200410 (PMC8995083; doi:10.1242/dev.200410)
Supplement: Reviewer comments [file develop-149-200410_review_history.pdf]

SCARECROW is deployed in distinct contexts during rice and maize leaf development

Thomas E. Hughes and Jane A. Langdale
DOI: 10.1242/dev.200410

Editor: Yka Helariutta

Review timeline
Original submission: 29 November 2021
Editorial decision: 14 January 2022
First revision received: 2 February 2022
Accepted: 28 February 2022

Original submission

First decision letter

MS ID#: DEVELOP/2021/200410

MS TITLE: SCARECROW is deployed in distinct developmental contexts during rice and maize leaf development

AUTHORS: Thomas E Hughes and Jane A Langdale

I have now received all the referees' reports on the above manuscript, and have reached a decision. The referees' comments are appended below, or you can access them online: please go to BenchPress and click on the 'Manuscripts with Decisions' queue in the Author Area.

As you will see, the referees express considerable interest in your work, but have some significant criticisms and recommend a substantial revision of your manuscript before we can consider publication. If you are able to revise the manuscript along the lines suggested, which may involve further experiments, I will be happy receive a revised version of the manuscript. Your revised paper will be re-reviewed by one or more of the original referees, and acceptance of your manuscript will depend on your addressing satisfactorily the reviewers' major concerns. Please also note that Development will normally permit only one round of major revision.

We are aware that you may be experiencing disruption to the normal running of your lab that make experimental revisions challenging. If it would be helpful, we encourage you to contact us to discuss your revision in greater detail. Please send us a point-by-point response indicating where you are able to address concerns raised (either experimentally or by changes to the text) and where you will not be able to do so within the normal timeframe of a revision. We will then provide further guidance. Please also note that we are happy to extend revision timeframes as necessary.

Please attend to all of the reviewers' comments and ensure that you clearly highlight all changes made in the revised manuscript. Please avoid using 'Tracked changes' in Word files as these are lost in PDF conversion. I should be grateful if you would also provide a point-by-point response detailing how you have dealt with the points raised by the reviewers in the 'Response to Reviewers' box. If you do not agree with any of their criticisms or suggestions please explain clearly why this is so.

© 2022. Published by The Company of Biologists under the terms of the Creative Commons Attribution License (https://creativecommons.org/licenses/by/4.0/).

Reviewer 1

Advance summary and potential significance to field

The authors use CRISPR/Cas9-mediated gene editing to show that two closely related SCR homologs in rice (OsSCR1 and OsSCR2) redundantly control internal root tissue layer patterning as is the case in maize and Arabidopsis. The genes' roles in the leaf, however, have diverged when compared to the C4 crop model maize. In maize the two SCR homologs regulated inner leaf tissue development, namely the number of mesophyll cells and enforcement of a single layer of bundle sheath cells around veins. In rice, inner leaf tissues seem unaffected yet a prominent role in stomatal development was described as leaves that developed postembryonically completely lack stomatal complexes. This shows that the role of the SCR genes albeit conserved in root patterning has diverged during leaf development even within the Poaceae.

Comments for the author

The data presented is convincing, the figures are clear and the manuscript is well written and structured. I only have one major concern/request and a couple of minor ones.

Major request

1. Increasing phenotypic penetrance in the leaves and qPCR The increasing phenotypic penetrance regarding the absence of stomatal complexes from leaf 3 to leaf 5 is very intriguing and I feel that the authors missed an opportunity to link expression levels of SCR1 and SCR2 in different leaves to the severity of the phenotype. I would expect that either the expression level of SCR1 and SCR2 change or that a closely related, partially complementing gene is not expressed in post-embryonic leaves anymore (e.g. Os07g38030, ZmSCR3?). Therefore a leaf number resolved qPCR of wild-type seedlings might be warranted here.
Similarly, I am not quite convinced by the qPCR data in Figure 5, particularly since there is only a marginal difference for OsFAMA, which is hard to explain when stomatal complexes are reduced and there is a clear reduction in OsMUTE. If I understood correctly, then whole seedling shoots at 4dag and 6dag were used for RNA extraction. I am not familiar with the exact onset of leaf development in rice, but I doubt that the 5th leaf is developing at this time point. So ideally RNA should be extracted and gene expression tested in developing leaf zones of the leaves that were assessed for stomatal phenotypes (leaf 3-5).

Minor concerns

2. Gene names: SCR1h or SCR2 While I do realize that Wu et al named the genes studied here SCR1 and SCR2, I think it is a bit unfortunate that OsSCrR1 and OsSCR2 rather correspond to ZmSCR1 and ZmSCR1h rather than ZmSCR2. I am not sure if this still can be changed.
Alternatively, it might be worthwhile to show the phylogenetic tree from Hughes et al. 2019 again here and indicate the rice and maize gene names to prevent confusion.
3. ZmSCR1 and ZmSCR1h expression patterns It is repeatedly stated that ZmSCR1 and ZmSCR1h are not expressed in the stomatal lineages (e.g. 2.6 paragraph 1 and discussion last paragraph). I am not sure however, that this has been unequivocally shown.
4. SCR in maize I think the fact that SCR seems to have such a distinct role in maize compared to rice leaves is very intriguing and I very much appreciate the detailed discussion the authors present. I would argue however that the SCR module might be fully occupied by patterning the inner leaf tissue architecture in C4 grasses and that the non-integration of stomata and vein patterning is a mere consequence of that.
In addition, it could be that the gene redundancy space in maize and rice are different and that ZmSCR2 could act redundantly to ZmSCR1 and ZmSCR1h in maize stomatal development, which might not be the case in rice. Again, information on the expression levels of OsSCR1 and OsSCR2 in different leaves might give additional evidence of whether this is the case or not.
5. References Please add references to the third paragraph of the Introduction after the sentence "In monocots, where stomata develop in rows flanking parallel veins, once the stomatal cell file is established cells divide asymmetrically to form a larger interstomatal sister cell and a GMC" -> Stebbins and Shah, 1960, McKown 2020 Nunes, 2020). Ideally, McKown 2020 and Nunes 2020 are added after the next sentence, too.

© 2022. Published by The Company of Biologists under the terms of the Creative Commons Attribution License (https://creativecommons.org/licenses/by/4.0/).

Reviewer 2

Advance summary and potential significance to field

The plant specific family transcription factor SCARECROW is known to play an essential role in endodermis specification in Arabidopsis and maize roots, bundle sheath development in Arabidopsis, and mesophyll cell patterning in maize. In rice, two duplicated SCR homologs have been previously implicated in stomata patterning, but it was unclear whether they are also required for inner leaf cell patterning. To answer this question, this study created double mutant for Osscr1 and Osscr2, and examined its leaf anatomy. Their results showed the mutant does not affect cell patterning, but severely affected stomata development in leaves. Based on cell organization and auto fluorescence, they concluded that the endodermis and exodermis are lost in the double mutant. The also conclude that SCR may regulate entry into the stomatal specification pathway. The authors also examined the stomata pattern in the maize Zmscr1;Zmscr1h double mutant but found not abnormality, making them to draw the conclusion that SCR play divergent roles in rice and maize.

This study broadens our understanding of the role played by SCARECROW in root and leaf development.

Comments for the author

Overall, the results are interesting and support their main conclusion. There are some issues that need to be clarified, as described below. Also, the description is a little dense in some parts that need to be clarified.

Main points:
1. In Fig 2: In addition to the endodermis and exodermis, the outmost cell layer in the stele appeared to be affected as well in both Osscr1;Osscr2 double mutants, is this true. If so, state it. Also, the fluorescence in the cortex is of different intensity, and form the wildtype. Does this means that the cortex is also affected?
2. In Fig 5: The figure legends are very hard to read. What is the meaning of the data points (circles)? I guess they are separate PCR result. If so, why are there so much variation? and why are there so much variation? Are they technical or biological replicates?
3. Based on the observation that OsMUTE is downregulated in the Osscr1 Osscr2 double mutant, wihile OsFAMA and OsROC5 are not affected, the authors concluded that “ SCR may regulate entry into the stomatal specification pathway.” If this is true, SCR should affect stomata development similarly in all leaves, but this is not the case. How to explain the observation that later leaves are affected more?
Along the same line, how to explain the observation that stomata development affected only on the adaxial sides of maize leaves, and no difference between embryonic leaves and true leaves?

Minor points:
1. “Although ROC5 levels appeared to be slightly reduced in the Osscr1-m7;Osscr2-m3 line, this was not significant;” Also, “In this comparison, ROC5 levels were slightly higher (though not statistically so) than in the mutant”. If not significant, how can one be sure that there is a difference?
2. It was stated that the Osscr1;Osscr2 double mutant form fewer and shorter roots than wildtype (Fig. 2A-F), it is better to show quantification analysis.

Reviewer 3

Advance summary and potential significance to field

See below

Comments for the author

Hughes and Langdale examine the function of the OsSCR1 and OsSCR2 genes from rice. They generate Osscr1 and Osscr2 loss of function lines using CRISPR and present the results of phenotypic

© 2022. Published by The Company of Biologists under the terms of the Creative Commons Attribution License (https://creativecommons.org/licenses/by/4.0/).

characterization of the single and double (Osscr1;Osscr2) mutants. This work follows well their previous papers examining the function of SCR in maize and their long-standing work on leaf vascular patterning in grass species. Surprisingly, they show a divergence in the function of SCR in rice and maize and a critical role for SCR in regulating MUTE and FAMA expression in the development of stomatal in rice. Overall, this was a very easy to read paper that is well focused and relevant. In reading the paper I had a lot of questions (e.g. what are the identities of the cells types in root? Does the root make aerenchyma?), which are probably beyond the scope of this nicely focused paper. However, other questions regarding leaf epidermal cell types could be addressed. For example – are there changes in the numbers or organization of the bulliform cells (the leaf rolling phenotype make this an obvious question). Are the stomata the only cell type affected? Are there changes in the number of hair cells, silica cells? Also I wonder if they examined STOMATOGEN expression and considered revising the pathway/model presented by Front. Plant Sci., 11 February 2020 | https://doi.org/10.3389/fpls.2019.01783

First revision

Author response to reviewers' comments

Response to reviewers

Reviewer 1 Advance Summary and Potential Significance to Field:

The authors use CRISPR/Cas9-mediated gene editing to show that two closely related SCR homologs in rice (OsSCR1 and OsSCR2) redundantly control internal root tissue layer patterning as is the case in maize and Arabidopsis. The genes' roles in the leaf, however, have diverged when compared to the C4 crop model maize. In maize, the two SCR homologs regulated inner leaf tissue development, namely the number of mesophyll cells and enforcement of a single layer of bundle sheath cells around veins. In rice, inner leaf tissues seem unaffected yet a prominent role in stomatal development was described as leaves that developed postembryonically completely lack stomatal complexes. This shows that the role of the SCR genes albeit conserved in root patterning has diverged during leaf development even within the Poaceae.

Reviewer 1 Comments for the Author:

The data presented is convincing, the figures are clear and the manuscript is well written and structured. I only have one major concern/request and a couple of minor ones.

Major request

1. Increasing phenotypic penetrance in the leaves and qPCR The increasing phenotypic penetrance regarding the absence of stomatal complexes from leaf 3 to leaf 5 is very intriguing and I feel that the authors missed an opportunity to link expression levels of SCR1 and SCR2 in different leaves to the severity of the phenotype. I would expect that either the expression level of SCR1 and SCR2 change or that a closely related, partially complementing gene is not expressed in post-embryonic leaves anymore (e.g. Os07g38030, ZmSCR3?). Therefore, a leaf number resolved qPCR of wild-type seedlings might be warranted here.

We thank the reviewer for this suggestion and have given it some thought. We too found the increasing penetrance of the phenotype intriguing. Little is known about the different patterning mechanisms that may operate in embryonic vs non-embryonic leaves. We agree that a leaf number resolved qPCR could shed some light on this, however, in practice we are not sure how feasible this would be since *SCR1* and *SCR2* are primarily expressed early in leaf development, and thus obtaining separate RNA samples would involve extremely challenging dissections of developing leaf primordia. The experiment probably needs to wait until spatial transcriptomics is feasible in rice.

© 2022. Published by The Company of Biologists under the terms of the Creative Commons Attribution License (https://creativecommons.org/licenses/by/4.0/).

We agree with the reviewer that a partially complementing gene could be expressed in a way that modifies the phenotype in different leaves, and have added this point to our discussion. We do not think this gene is likely to be the rice ortholog of ZmSCR2/AtSCL23 Os07g38030 as in transcriptome data it is not co-expressed with OsSCR1 and OsSCR2 (which peak at P3), and is instead expressed more highly at P5, after stomatal differentiation (van Campen *et al*. 2016). Furthermore, a preliminary look at a maize *Zmscr1;Zmscr1h;Zmscr2* triple mutant did not reveal any additional patterning phenotypes (at least not in inner leaf tissues). We feel that untangling further redundancy between closely related SCR genes is a relevant topic for further investigation, but outside of the scope of this study.

Similarly, I am not quite convinced by the qPCR data in Figure 5, particularly since there is only a marginal difference for OsFAMA, which is hard to explain when stomatal complexes are reduced and there is a clear reduction in OsMUTE. If I understood correctly, then whole seedling shoots at 4dag and 6dag were used for RNA extraction. I am not familiar with the exact onset of leaf development in rice, but I doubt that the 5th leaf is developing at this time point. So ideally, RNA should be extracted and gene expression tested in developing leaf zones of the leaves that were assessed for stomatal phenotypes (leaf 3-5).

We thank the reviewer for this feedback as it has helped us in our interpretation of the qPCR data. At the initial 6 days after sowing timepoint sampled, growth kinetic analyses show that leaf 3 is normally at the P4 stage, leaf 4 at P3 and leaf 5 at P2. *OsSCR1* and *OsSCR2* transcript levels are highest at P3, stomatal patterning happens during P3 and stomatal differentiation during P4 (van Campen *et al*. 2016). As such, because leaf 4 is at the stomatal patterning stage at the time of sampling and very few stomata develop in the mutant, transcript levels of the patterning gene *OsMUTE* are reduced in mutants. In contrast, leaf 3 is at the stomatal differentiation stage at the time of sampling and because stomata develop on that leaf in mutants, transcript levels of the differentiation associated gene *OsFAMA* are less affected than *OsMUTE*. We have amended the discussion to explain this, and thank the reviewer for drawing our attention to it.

Minor concerns

2. Gene names: SCR1h or SCR2 While I do realize that Wu et al named the genes studied here SCR1 and SCR2, I think it is a bit unfortunate that OsSCrR1 and OsSCR2 rather correspond to ZmSCR1 and ZmSCR1h rather than ZmSCR2. I am not sure if this still can be changed. Alternatively, it might be worthwhile to show the phylogenetic tree from Hughes et al. 2019 again here and indicate the rice and maize gene names to prevent confusion.

We agree with the reviewer that the naming of orthologous SCR genes has become confusing in the literature. ZmSCR1h was named as such due to it being the homeolog copy from the maize whole genome duplication. Most maize homeolog genes are single copy in rice since they did not undergo the same recent whole genome duplication. Surprisingly, however, SCR has independently duplicated in rice, *Setaria* and sorghum. Because the maize ortholog of AtSCL23 had already been named ZmSCR2, which we felt made sense due to it being the most closely related gene to ZmSCR1 and ZmSCR1h, we can see that it is confusing that the rice genes are named here OsSCR1 and OsSCR2. However, we felt that it was more important to keep the same gene names as had been used by Wu et al (2019) to avoid adding further confusion to the literature. We have taken the authors suggestion and included a cartoon depiction of our 2019 phylogeny as a new figure 1 in the paper.

3. ZmSCR1 and ZmSCR1h expression patterns

It is repeatedly stated that ZmSCR1 and ZmSCR1h are not expressed in the stomatal lineages (e.g. 2.6 paragraph 1 and discussion last paragraph). I am not sure, however, that this has been unequivocally shown.

We thank the reviewer for this point and have softened the language to reflect this uncertainty.

4. SCR in maize

© 2022. Published by The Company of Biologists under the terms of the Creative Commons Attribution License (https://creativecommons.org/licenses/by/4.0/).

I think the fact that SCR seems to have such a distinct role in maize compared to rice leaves is very intriguing and I very much appreciate the detailed discussion the authors present. I would argue however that the SCR module might be fully occupied by patterning the inner leaf tissue architecture in C4 grasses and that the non-integration of stomata and vein patterning is a mere consequence of that. In addition, it could be that the gene redundancy space in maize and rice are different and that ZmSCR2 could act redundantly to ZmSCR1 and ZmSCR1h in maize stomatal development, which might not be the case in rice. Again, information on the expression levels of OsSCR1 and OsSCR2 in different leaves might give additional evidence of whether this is the case or not.

We are pleased that the reviewer found the discussion helpful. We agree that altered redundancy with other genes could underpin the differences found between species, and have added a section to the discussion considering this. We do not agree with the reviewer that the SCR module could be ‘fully occupied’ in the inner-leaf in C4 grasses, since there is no inherent reason why SCR could not be deployed in both stomatal and inner-leaf patterning in the same species.

5. References

Please add references to the third paragraph of the Introduction after the sentence "In monocots, where stomata develop in rows flanking parallel veins, once the stomatal cell file is established cells divide asymmetrically to form a larger interstomatal sister cell and a GMC" -> Stebbins and Shah, 1960, McKown 2020, Nunes, 2020). Ideally, McKown 2020 and Nunes 2020 are added after the next sentence, too.

We thank the reviewer for this suggestion and have added the references.

Reviewer 2 Advance Summary and Potential Significance to Field: The plant specific family transcription factor SCARECROW is known to play an essential role in endodermis specification in Arabidopsis and maize roots, bundle sheath development in Arabidopsis, and mesophyll cell patterning in maize. In rice, two duplicated SCR homologs have been previously implicated in stomata patterning, but it was unclear whether they are also required for inner leaf cell patterning. To answer this question, this study created double mutant for Osscr1 and Osscr2, and examined its leaf anatomy. Their results showed the mutant does not affect cell patterning, but severely affected stomata development in leaves. Based on cell organization and auto fluorescence, they concluded that the endodermis and exodermis are lost in the double mutant. The also conclude that SCR may regulate entry into the stomatal specification pathway. The authors also examined the stomata pattern in the maize Zmscr1;Zmscr1h double mutant but found not abnormality, making them to draw the conclusion that SCR play divergent roles in rice and maize.

This study broadens our understanding of the role played by SCARECROW in root and leaf development.

Reviewer 2 Comments for the Author:

Overall, the results are interesting and support their main conclusion. There are some issues that need to be clarified, as described below. Also, the description is a little dense in some parts that need to be clarified.

Main points:

1. In Fig 2: In addition to the endodermis and exodermis, the outmost cell layer in the stele appeared to be affected as well in both Osscr1;Osscr2 double mutants, is this true. If so, state it. Also, the fluorescence in the cortex is of different intensity, and form the wildtype. Does this means that the cortex is also affected?

We agree that the differing fluorescence in the cortex and outer cell-layers of the roots could indicate an effect on these cell-types in the mutant. However, we have only highlighted the

© 2022. Published by The Company of Biologists under the terms of the Creative Commons Attribution License (https://creativecommons.org/licenses/by/4.0/).

altered endodermis and exodermis as they are most obvious and consistent across the samples analysed. Further root patterning effects will be a productive route for future investigation.

2. In Fig 5: The figure legends are very hard to read. What is the meaning of the data points (circles)? I guess they are separate PCR result. If so, why are there so much variation? and why are there so much variation? Are they technical or biological replicates?

Data points are biological replicates, hence variation is between different plants sampled. There is inherent variability with regards to leaf development in rice at this developmental stage, and as such we do not believe it is surprising to see variation between individuals of the same genotype. However, we think the clear and consistent differences between genotypes, at least in the case of MUTE, is compelling evidence that MUTE is downregulated in *Osscr1;Osscr2* mutants.

We were not sure what the reviewer meant by the figure legends were hard to read, whether unclear, hard to find etc. but have tried to ensure their clarity.

3. Based on the observation that OsMUTE is downregulated in the Osscr1 Osscr2 double mutant, wihile OsFAMA and OsROC5 are not affected, the authors concluded that " SCR may regulate entry into the stomatal specification pathway." If this is true, SCR should affect stomata development similarly in all leaves, but this is not the case. How to explain the observation that later leaves are affected more?

We thank the reviewer for this comment, and agree that we have not revealed the mechanism by which the penetrance of the phenotype increases from leaf 3 to 5. We have amended the statement to 'SCR may regulate entry into the stomatal specification pathway, particularly in non-embryonic leaves', and have also included some discussion of whether altered expression levels of either SCR1 and SCR2 or a closely related gene in different leaves may underpin these differences.

Along the same line, how to explain the observation that stomata development affected only on the adaxial sides of maize leaves, and no difference between embryonic leaves and true leaves?

We agree with the reviewer that these observations in maize are intriguing. At the simplest level we think they support our conclusion that there is extensive divergence in SCR function between rice and maize, in that there is no abaxial reduction in stomatal density in maize, nor any change between embryonic and non-embryonic leaves, both of which were seen clearly in rice. There are a number of possible explanations for the reduction of stomatal density on the adaxial surface of maize leaves. For example, it is possible that SCR retains some stomatal function in maize, specifically on the adaxial surface, but that its primary function is to regulate inner-leaf patterning. It is also possible that these differences reflect an environmental or physiological effect of the reduced growth of maize *scr* mutants, and not a direct patterning role. In support of this, we believe SCR is primarily expressed in the inner- leaf in maize rather than in developing stomata. However, the recent finding that SCR is mobile in maize roots (Ortiz-Ramirez et al. 2021) means a non-cell autonomous patterning function is also plausible. These points have been reinforced in the relevant 2.6 results section.

Minor points:

"Although ROC5 levels appeared to be slightly reduced in the Osscr1-m7;Osscr2-m3 line, this was not significant;" Also, "In this comparison, ROC5 levels were slightly higher (though not statistically so) than in the mutant". If not significant, how can one be sure that there is a difference?

One cannot be sure of subtle differences in small sample sizes, which we were trying to reflect in our language. Regardless, any possible difference in ROC5 levels does not affect the primary conclusion that OsMUTE was downregulated in *Osscr1;Osscr2* mutants. We have amended the language to reflect the lack of statistically significant difference.

1. It was stated that the Osscr1;Osscr2 double mutant form fewer and shorter roots than wildtype (Fig. 2A-F), it is better to show quantification analysis.

© 2022. Published by The Company of Biologists under the terms of the Creative Commons Attribution License (https://creativecommons.org/licenses/by/4.0/).

We thank the author for this suggestion. We do not have quantitative analysis of root numbers but believe the presence of shorter roots is obvious from the photos so have amended the statement to read 'double mutants form shorter roots than wildtype'.

Reviewer 3 Advance Summary and Potential Significance to Field:

See below

Reviewer 3 Comments for the Author:

Hughes and Langdale examine the function of the OsSCR1 and OsSCR2 genes from rice. They generate Osscr1 and Osscr2 loss of function lines using CRISPR and present the results of phenotypic characterization of the single and double (Osscr1;Osscr2) mutants. This work follows well their previous papers examining the function of SCR in maize and their long- standing work on leaf vascular patterning in grass species. Surprisingly, they show a divergence in the function of SCR in rice and maize and a critical role for SCR in regulating MUTE and FAMA expression in the development of stomatal in rice. Overall, this was a very easy to read paper that is well focused and relevant. In reading the paper I had a lot of questions (e.g. what are the identities of the cells types in root? Does the root make aerenchyma?), which are probably beyond the scope of this nicely focused paper. However, other questions regarding leaf epidermal cell types could be addressed. For example - are there changes in the numbers or organization of the bulliform cells (the leaf rolling phenotype make this an obvious question). Are the stomata the only cell type affected? Are there changes in the number of hair cells, silica cells? Also I wonder if they examined STOMATOGEN expression and considered revising the pathway/model presented by Front. Plant Sci., 11 February 2020 | https://doi.org/10.3389/fpls.2019.01783

We thank the reviewer for their comments and are pleased they enjoyed the paper. We agree that there are further questions to be addressed in relation to the root patterning phenotype, and that those questions are beyond the scope of this paper. We agree that analysing the formation of the bulliform cells in our cross sections is worthwhile, given the leaf rolling phenotype, and we present a quantitative analysis of bulliform cell number in a new supplemental figure. No statistically significant reduction in the % of bulliform cells that comprise the epidermis was found, indicating that OsSCR does not pattern bulliform cells in a similar manner to stomata. However, an effect on bulliform cell shape and/or size may lead to impaired function and underpin the leaf rolling phenotype.

Second decision letter

MS ID#: DEVELOP/2021/200410

MS TITLE: SCARECROW is deployed in distinct contexts during rice and maize leaf development

AUTHORS: Thomas E Hughes and Jane A Langdale
ARTICLE TYPE: Research Article

I am happy to tell you that your manuscript has been accepted for publication in Development, pending our standard ethics checks.

Reviewer 1

Advance summary and potential significance to field

see below

Comments for the author

© 2022. Published by The Company of Biologists under the terms of the Creative Commons Attribution License (https://creativecommons.org/licenses/by/4.0/).

First, I very much appreciate that the authors included a cartoon phylogeny but don't quite understand why they omitted ZmSCR2/AtSCL23 branch. Particularly, since SCL23 comes up in the discussion.
Second, their explanation regarding FAMA and MUTE transcript levels being related to the sampling stage seems very plausible. Yet, since it seems to be very clear at which day after germination which plastochron is developing, I don't quite understand, why the authors did not perform an additional qPCR experiment at a later timepoint (8-10dag). This would show that their hypothesis regarding FAMA and MUTE transcript levels is indeed true (8-10dag). In addition, this material could be used to assess if SCR1 and SCR2 levels are changing towards later timepoints. That being said, I am not convinced that these experiments really add additional strength to the author's main conclusions, but still think that is a missed opportunity.

Reviewer 2

Advance summary and potential significance to field

This study broadens our understanding of the role played by SCARECROW in root and leaf development.

Comments for the author

My questions have been largely addressed. I still think the figure legends for Fig 5 can be improved. For example, what is the meaning of the data points(circles), which is a very specific question.

Reviewer 3

Advance summary and potential significance to field

As stated in original review

Comments for the author

As indicated in previous review, delving into a characterization of the root phenotype of these plants seems outside of the scope of the paper. Examination of STOMATOGEN expression however is central to the network they are examining as is considered of the model presented by Front. Plant Sci., 11 February 2020 | https://doi.org/10.3389/fpls.2019.01783, which is not even cited in the paper.

© 2022. Published by The Company of Biologists under the terms of the Creative Commons Attribution License (https://creativecommons.org/licenses/by/4.0/).
